# Social image concerns promote cooperation more than altruistic punishment

**DOI:** 10.1038/ncomms12288

**Published:** 2016-08-09

**Authors:** Gianluca Grimalda, Andreas Pondorfer, David P. Tracer

**Affiliations:** 1 Institute for the World Economy, Kiellinie 66, 24105 Kiel, Germany; 2 Universitat Jaume I, Avenida Sos Baynat, 12071 Castelló de la Plana, Spain; 3 Centre for Global Cooperation Research, University of Duisburg-Essen, Schifferstrasse 196, 47059 Duisburg, Germany; 4 Department of Health and Behavioral Sciences, University of Colorado Denver, Campus Box 188, PO Box 173364, Denver, Colorado 80217-3364, USA

## Abstract

Human cooperation is enigmatic, as organisms are expected, by evolutionary and economic theory, to act principally in their own interests. However, cooperation requires individuals to sacrifice resources for each other's benefit. We conducted a series of novel experiments in a foraging society where social institutions make the study of social image and punishment particularly salient. Participants played simple cooperation games where they could punish non-cooperators, promote a positive social image or do so in combination with one another. We show that although all these mechanisms raise cooperation above baseline levels, only when social image alone is at stake do average economic gains rise significantly above baseline. Punishment, either alone or combined with social image building, yields lower gains. Individuals' desire to establish a positive social image thus emerges as a more decisive factor than punishment in promoting human cooperation.

## Abstract (web summary)

Cooperation requires individuals to sacrifice individual rewards for group benefits. Here, Grimalda, Pondorfer and Tracer show in a foraging society of Papua New Guinea that social image building is a more powerful motivator of social cooperation than altruistic punishment.

## Graphical abstract

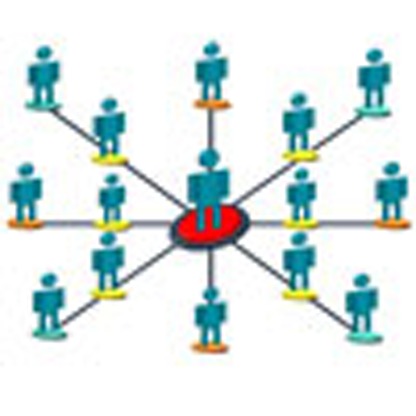

According to evolutionary and economic theories, humans, similar to other animals, are expected to behave selfishly, maximizing material gains for themselves^1^,^2^,^3^. Nevertheless, human cooperation occurs in all known societies and is common between genetically unrelated individuals and where repeated interactions may be uncommon. This is enigmatic and demands explanation^3^. Several mechanisms have been proposed to explain the evolution of cooperation^4^. Among them, indirect reciprocity has attracted the attention of many researchers^5^,^6^,^7^,^8^. Indirect reciprocity can take many forms, although one that seems to be particularly effective for cooperation builds on an individual's desire to maintain a reputation as a cooperator in the social group. Reputation-building is possible, because information about an individual's cooperative behaviour in social interactions diffuse through the social network. Others can then use such information to either extend cooperation back to a cooperator or refrain from doing so vis-à-vis a defector. Evolutionary models assume that individuals accrue image scores based on the frequency with which they cooperated in the past. An individual has an incentive to maintain a reputation as a cooperator, because in future interactions she will in turn benefit from others' cooperation in the group. An extensive body of empirical and experimental evidence confirms that maintaining a reputation as a cooperator indeed commands material rewards from other group members^9^,^10^,^11^. Compared with direct reciprocity^12^, indirect reciprocity expands the scope of cooperation, because an altruistic action will be reciprocated not only by the direct beneficiary of the action but also by whomever has knowledge of the cooperator's positive social image.

An alternative account singles out costly punishment as a factor that can also increase cooperation. This relies on individuals' propensity to punish, even at a cost to themselves, people who defect from cooperation or who deviate from norms of fairness^13^,^14^,^15^. Such punishment has been frequently observed in ephemeral ‘one-shot' interactions, or is performed by ‘third parties'^16^,^17^,^18^,^19^. As in both cases the punisher cannot receive any material gain from punishment, this has been labelled ‘altruistic'^20^. Punishment appears widespread in human groups ranging from small-scale traditional societies to large-scale complex societies^17^. It has been argued that altruistic punishment is not a mechanism for the evolution of cooperation *per se*, but rather is a proximate factor that can enhance cooperation when latched onto by other evolutionary mechanisms including direct or indirect reciprocity, or group selection^4^,^21^.

There is however a profound disavantage to using altruistic punishment to maintain cooperation; because punishment entails a cost both for the punisher and the punished, it is expensive and inefficient, resulting in considerable monetary losses^22^,^23^,^24^ or, at worst, destructive acts of vengeance^25^,^26^. Moreover, the act of punishment entails a second-order public goods problem whose solution may require strategic coordination, asymmetries or the insurgence of a centralized authority^27^,^28^. By contrast, building a positive social image is potentially a cheaper, automatic and more efficient means of enforcing cooperation^22^,^29^,^30^. Arguably, maintaining a positive social image is motivationally less demanding than altruistic punishment and furnishes a different basis for the moral norms that become established in a community. Rather than relying on altruistic motivations, maintaining a good social image is in the individual's long-term self-interest^13^. The main drawback of reputational mechanisms is that they are only effective in communities where there is high ‘broadcast efficiency', that is, reliable information about individuals' past behaviours can diffuse rapidly through the community^31^. We expect this to be most common in small, tight-knit communities where gossip travels quickly or where there exists an authority or institution that occupies a central position amid its social networks and serves as a conduit for disseminating information about people's social images. Nonetheless, observability of one's actions can have extensive consequences even in contemporary societies^32^ and it has been argued that online reputation systems and social media have extended broadcast efficiency even to large populations^33^. In addition, individuals are likely to be much more concerned about their social image in the presence of other members of their group rather than outsiders^34^,^35^,^36^. Indeed, preferential concern for social image among ingroup compared with outgroup members and the need to rapidly assess group membership may have favoured the elaboration of ethnic markers in, for instance, dress, dialect and bodily adornment over the course of human evolutionary history^37^.

Social image and altruistic punishment are not mutually exclusive motivators of cooperation, but may be activated, separately or jointly, in different situations^38^. It is likely to be that cultural factors and specific social structures may favour one or the other factor. It is nonetheless interesting to investigate their relative efficacy in promoting social welfare, to shed light on the respective role that they can play to sustain human cooperation. The evidence on this topic is scant and limited to few experiments where one's social image is artificially created and maintained in the laboratory^22^,^39^,^40^,^41^. Moreover, these studies were conducted in Western, industrialized contexts and among strangers where reputation effects are ephemeral. A compelling argument has been made^42^ that these conditions are relatively novel within evolutionary history, and that data from non-Western small-scale societies are essential for testing hypotheses about human psychology, especially in the domains of preferences and decision-making.

Here we provide data from the first-ever study of the relative roles of indirect reciprocity and alruistic punishment, alone and in combination, in promoting cooperation among the Teop, a small-scale society located in Bougainville, Papua New Guinea. In doing so, we are among the first to study the effects on cooperation of real-life social image rather than artificial, experimentally created, social image^33^,^43^,^44^,^45^. We do so by using persons of authority as observers. Social relationships in Teop revolve around the figure of the ‘Big Man' (BM). Big men possess exclusive knowledge and ‘impose discipline, uphold the traditional way of life and give executive directions'^46^ to other community members. Social disputes or problems of coordination between clans are normally dealt with under the supervision or explicit intervention of Big Men. Big Men have informal authority and also act as ‘guardians of morality' within the society. Arguably, they are figures towards whom individuals strive to keep a positive social image. Moreover, they are ‘hubs' of the social network; hence, they are central in disseminating social image information through the society. Running our study in Teop gives us the opportunity to examine the impact of one's social image in a context presumably closer to that characterizing human societies for the majority of our evolutionary history^35^.

Our experiments contrast concerns for social image vis-à-vis the BM and punishment as factors promoting cooperation, either in isolation or combined. All these factors raise cooperation above baseline levels. Nevertheless, only when social image alone is at stake do average economic gains rise significantly above baseline. Punishment, either alone or combined with social image building, yields gains even lower than the baseline. We also show that when a BM from an external group acts as the observer, again payoffs do not rise above baseline. We conclude that individuals' desire to establish a positive social image within their community emerges as a more decisive factor than punishment in promoting human cooperation.

## Results

### The experimental game

We conducted a series of anonymous, one-shot, prisoner's dilemma (PD) games involving two participants. In the baseline condition, each participant received 10 Kina (K10) to be used in the game (Endowment I), plus K4 (Endowment II) that were not used in the game but were cashed in at the end of the game. Participants had to decide whether they wanted to keep K10, or give K10 to the other participant. If a participant kept the K10, this person would receive the K10 at the end of play. If a participant gave K10, the other person would receive K20 at the end of play because, as it was explained, the researcher would add K10 to the exchange. The payoff structure of the game, in its simplicity, resembles a ‘tragedy of the commons' scenario^4^,^5^,^9^. Mutual cooperation—namely, both players giving to the counterpart—ensures the highest payoff for the group, but mutual defection—namely, both players keeping their Endowment I—is the rational strategy for individual payoff maximization.

To test for effects of social image concerns and altruistic punishment in motivating cooperation, we implemented four additional experimental treatments (summarized in Table 1). In treatment ‘BM' participants played the PD as in the baseline, with the key difference that a BM from the same village as each participant was present in the room and observed his or her decision. In ‘Big Man External' (BM EXT), each decision was witnessed by a BM from a different ethno-linguistic group. These treatments capture differences in experimental outcomes when Big Men having clear and different social distances from the participants are present, as well as varying informal authority. The extant literature demonstrates that being observed by one's peers leads individuals to modify their behaviours in significant ways^43^,^44^,^45^,^47^, although this may lead to less pro-sociality when ingroup–outgroup relationships are made salient^36^.

We modelled altruistic punishment as is standard in the literature^18^,^20^,^22^,^23^,^25^ by introducing a punishment stage after all participants played the PD game described above. Either player had the option of spending the K4 from Endowment II to reduce the other participant's payoff. Each participant could spend K0, K2 or K4 to reduce the counterpart's payoff by K0, K10 or K20, respectively. We used the ‘strategy method'^17^,^35^ to investigate punishment patterns. Each participant had to make two decisions under the assumption that the other participant had either kept K10 or given K10. This allows us to examine patterns of what have been named ‘altruistic' punishment—that is, punishment when the other player defects—and ‘anti-social' punishment—that is, punishment when the other player cooperates^25^,^30^. To study the interaction between social image concerns and altruistic punishment, we added a ‘Big Man+Punishment' (BM+PUN) treatment in which the local BM observed both the PD and punishment choices, in the same way as in the BM treatment.

Participants made their decisions privately and anonymously in all conditions, never knowing who was their co-player. The only information they received was that the other player was from either their own or a neighbouring village. Unlike other research conducted in small-scale societies^17^, experimenters and local assistants left the experimental room when participants made their choices. This was done to maximize the saliency of the BM alone, rather than the experimenters, in participants' concerns with social image building and their consequent choices. Handing out the K4 ‘Endowment II' in all treatments guarantees the absence of income effects across treatments (net of the punishment decision in the punishment treatments). Before participants made their decisions, they had to pass a thorough comprehension check (see Methods, Supplementary Methods sections 2.3 and 3.2, and Supplementary Table 1 for demographic characteristics of the sample).

### Cooperation is highest in the ‘BM' treatment

**Figure 1:**
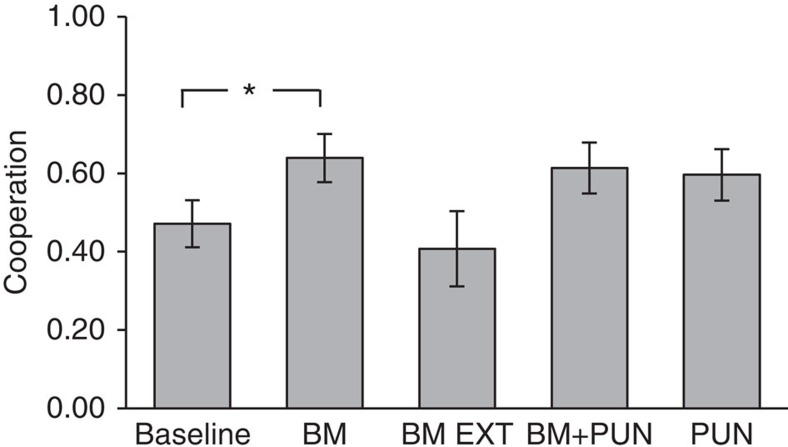
Mean cooperation rates by treatment. Error bars reflect ±1 s.e.m. Top horizontal bars show results of pairwise Wald's tests over the existence of significant treatment differences in a logit regression, as per model reported in Supplementary Table 3, column 3. *P<0.05. Only significant tests from such regressions analyses are reported. Cooperation rates are highest in the BM treatment (*n*=61). These are statistically significantly higher than those in baseline (*n*=70), while cooperation rates in BM EXT (*n*=27), BM+PUN (*n*=57) and PUN (*n*=57) are not.

Figure 1 plots mean cooperation rates per treatment. Cooperation is highest in the BM treatment. 63.9% of participants gave their Endowment I to their counterpart in the BM condition, whereas cooperation rates in PUN and BM+PUN are 3–4% lower than in BM. However, such cooperation rates are not statistically different from each other (Wald's tests derived from logit regression; *P*=0.60, *N*=272 for difference between BM and PUN; *P*=0.67, *N*=272 for difference between BM and BM+PUN; *P*=0.92, *N*=272 for difference between PUN and BM+PUN; all tests being reported are two-tailed; see Supplementary Tables 2 and 3 and ‘Statistical methods') in a logit model that controls for village effects, experimenter identity effects, gender and comprehension. The same conclusion holds if adding additional demographic controls for age, education and an index of household wealth (see Supplementary Tables 3 and 4), or if any control is omitted (see Supplementary Table 3, column 1). Among demographic controls, we note that age is positively associated with cooperation (*P*=0.018), which is in line with findings from Western societies^48^. Education, too, exerts a positive effect on cooperation (*P*=0.029 for ‘Years of Education' longer than 10 years).

47.1% of participants cooperated in the baseline condition. This is statistically significantly lower than BM (logit regression, *P*=0.024, *N*=272), although it falls short of statistical significance at conventional levels with respect to either PUN or BM+PUN (logit regression, *P*=0.077, *N*=272 for PUN; *P*=0.088, *N*=272 for BM+PUN). We conclude that the presence of BM is particularly effective in raising cooperation above the baseline.

### External BM does not bring about cooperative gains

If cooperation is truly motivated by participants' concerns about their social image, we expect cooperation in BM EXT to be lower than in BM because of the farther social distance between external Big Men and participants compared with local Big Men. In agreement with our expectation, cooperation is substantially lower in BM EXT (40.7%) than in BM (63.9%), although this difference does not reach statistical significance (logit regression, *P*=0.14, *N*=272, see Supplementary Table 3). Cooperation in BM EXT is in fact even marginally lower than in the baseline condition (logit regression, *P*=0.913, *N*=272).

We validated participants' beliefs about their social distance from either the external or the local Big Men using a post-experiment questionnaire (see Supplementary Discussion section 1.3). We show that a measure of closeness in the social network between participant and BM—as per the frequency of their past and future encounters—is a significant predictor of cooperation. On the contrary, both the acquaintance with the BM and the recognition of the legitimacy of the BM's guidance in every day's life have no impact (see Supplementary Tables 5–7). This helps qualify which motivations are more relevant in the willingness to maintain a positive social image with the BM and suggests that the closeness in the social network has a dominant influence for such motives.

### Punishment is less frequent in BM+PUN than in PUN treatment

Some researchers have posited that individuals will refrain from punishing when another's reputation is at stake, because indirect reciprocity mechanisms will ‘indirectly' punish the individual^22^,^49^,^50^. In our context, punishment should then be lower when the BM is present, because individuals anticipate that tarnishing one's social image by defecting in the presence of the BM is enough to enforce cooperation. Conversely, others would posit that punishment should be higher when the BM is present if individuals think that their social image will benefit from punishing a defector in the presence of the BM. According to this hypothesis, acquiring a ‘fierce' reputation for being a punisher may pay off in evolutionary terms^7^,^51^, because such a reputation commands either fear^52^ or reward^53^,^54^. Evidence in favour of both predictions has been found^17^,^49^,^52^,^53^,^54^,^55^. Our novel experimental design enables us to directly test these two alternative hypotheses.

**Figure 2:**
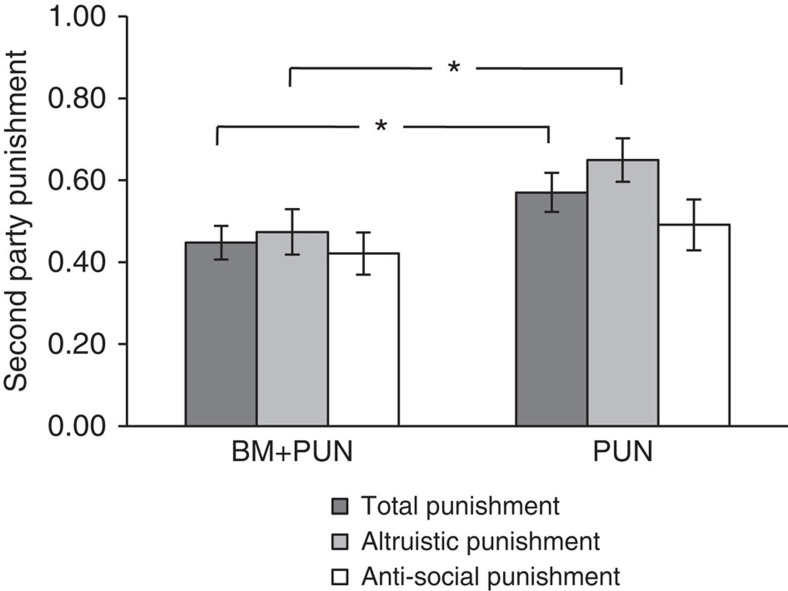
Fraction of Endowment II spent in punishment. Error bars reflect ±1 s.e.m. The white column represents the fraction of endowment spent for anti-social punishment. The light-shaded column represents the fraction of endowment spent for altruistic punishment. The darkest column is the average between the previous two punishment levels and thus represents total punishment. Top horizontal bars show results of Wald's tests over the existence of significant treatment differences between BM+PUN (*n*=57) and PUN (*n*=57) in an ordered logit regression, as per models reported in the Supplementary Table 9, columns 3, 7 and 11. *P<0.05. Both overall punishment and altruistic punishment—that is, punishment of defectors—is significantly higher in PUN than in BM+PUN.

In our study, punishment is significantly lower in BM+PUN than in PUN. Participants spent on average 57% of their Endowment II in PUN and 44.7% in PUN+BM (Fig. 2). This difference is statistically significant (ordered logit regression, *P*=0.039, *N*=228), in an ordered logit model similar to that used to study cooperation (see Supplementary Tables 8–9), and the result is robust to the inclusion of demographic controls. The presence of the BM appears particularly effective in restraining individuals from punishment when the other party has defected. In this case, the money spent for punishment is reduced by 27.3% (ordered logit regression, *P*=0.019, *N*=114; see Supplementary Table 9). If the other player cooperated, punishment costs were reduced by 14.3% (ordered logit regression, *P*=0.405, *N*=114). The result that punishment decreases significantly when the BM is present again supports our primary contention that social image concerns are paramount in social interactions and may ‘crowd out' motivations to engage in other, more costly forms of social control such as punishment.

Anti-social punishment is found in Teop at a level comparable with several other experiments conducted in various cultural areas^25^,^56^,^57^,^58^ and is substantially higher than what is found in most Western cultures^59^ (see Supplementary Discussion section 1.4 and Supplementary Fig. 1). In particular, a study conducted in Russia^56^ under a framework similar to ours found that as many as 55% of the most cooperative people were punished, whereas this fraction is only marginally higher (60%) in Teop (see Supplementary Fig. 2). Evolutionary biology models^30^,^60^,^61^,^62^ and other accounts^63^ can explain the occurrence of anti-social punishment. Although participants' confusion with the game may have played a part in anti-social punishing, we believe that this effect is marginal. We note that participants' comprehension was thoroughly assessed (see Supplementary Methods sections 2.3 and 3.2), and that a variable identifying participants' number of mistakes in such comprehension checks is never significant at conventional levels in our regression analysis (see in particular Supplementary Tables 3 and 9). Interestingly, overall punishment correlates negatively with the frequency of attendance at religious services (ordered logit regression, *P*=0.002, *N*=218; see Supplementary Table 10), regardless of religious affiliation. This shows that punishment behaviour rises with lower engagement in religious and communal life.

Anti-social punishment also emerged in some additional experimental sessions that we ran in which third parties, rather than the players involved in the PD, were given the option to punish a PD player (see Supplementary Methods sections 2.4 and 3.3 for details on the procedures). Punishment by third parties is clearly selective across the different PD outcomes (see Supplementary Fig. 3 and Supplementary Tables 11 and 12) and is directly proportional to the PD player's payoff (ordinary least-square regression, *P*<0.001, *N*=84; see Supplementary Table 13). This refutes the notion that confusion drove participants' behaviour. As third parties' payoffs were by design always greater than or equal to a PD-player's payoff, aversion to disadvantageous inequality^64^ can also be ruled out as a possible motivation. This leaves spite^57^,^58^,^63^,^65^ as a probable candidate to explain anti-social punishment in Teop. It is notable as mentioned above that regular participation in religious institutions may act to lower individuals' motivations to act spitefully towards others.

### Payoffs are maximized in the ‘BM' treatment

**Figure 3:**
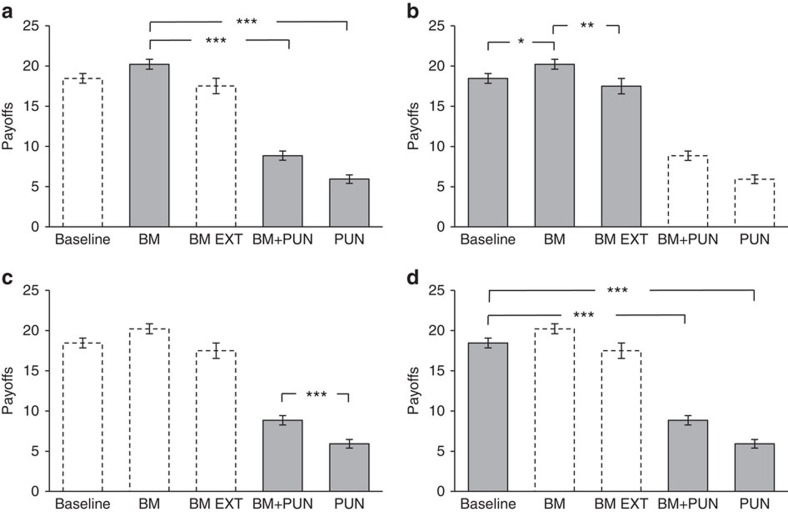
Average payoffs per treatment. Error bars reflect ±1 s.e.m. Top horizontal bars show results of pairwise Wald's tests over the existence of significant treatment differences in a Tobit regression (see Supplementary Table 14, column 3). *P<0.05, **P<0.01 and ***P<0.001. The four panels reproduce the same data, with shaded columns being relevant for the following results. (**a**) Payoffs in BM (*n*=61) are significantly larger than in BM+PUN (*n*=57) and PUN (*n*=57). (**b**) Payoffs in BM are significantly higher than in the baseline condition (*n*=70) and BM EXT (*n*=27). (**c**) Payoffs in PUN are significantly lower than in BM+PUN. (**d**) Payoffs in PUN and BM+PUN are significantly lower compared with baseline.

To compare the relative success of different mechanisms for enforcing cooperation, the key variable of interest is not average cooperation *per se*, but rather the average payoff^22^,^23^,^39^,^41^,^66^. Figure 3 reports the average payoff per treatment. In the two punishment treatments we report the payoff from the PD game net of the average punishment costs sustained by both the punisher and the punished. As is standard in recent evolutionary analyses of altruistic punishment and indirect reciprocity^6^,^7^,^14^,^15^, payoffs are determined as those resulting from the actions actually performed by the participant combined with the average cooperation and punishment rates observed in the same treatment where the participant has been involved.

Payoffs are conspicuously larger in the BM treatment (K20.2) than in either the BM+PUN or PUN treatments (Fig. 3a; Tobit regression; *P*<0.001, *N*=272, for BM+PUN; *P*<0.001, *N*=272 for PUN; see Supplementary Tables 2 and 14, and Supplementary Fig. 4). In fact, the BM treatment is the only treatment where average payoffs exceed the baseline (Fig. 3b; Tobit regression, *P*=0.016, *N*=272). Payoffs in BM are also significantly higher than in BM EXT (Fig. 3b; Tobit regression; *P*=0.006, *N*=272). The comparison between BM and BM+PUN is particularly interesting. In both treatments the BM is present; thus, social image concerns are relevant for participants in both conditions. However, it is apparent that the interaction between social image and altruistic punishment is not efficient relative to BM alone. That is, introducing punishment in the presence of social image concerns is detrimental to payoffs.

Even if BM+PUN is more efficient than PUN, it is nevertheless striking that in both cases the average payoffs are lower than the baseline case. The difference is statistically significant for both PUN and BM+PUN treatments compared with baseline (Fig. 3d; Tobit regression, *P*<0.001, *N*=272 for PUN; *P*<0.001, *N*=272 for BM+PUN). We therefore conclude that the introduction of punishment devices is overall detrimental, and that social image concerns, as manifested in our BM treatment, are paramount in promoting cooperation and the general social welfare.

## Discussion

We find that concerns about social image, here manifested by the actions that one takes in a one-shot PD game in the presence of a local authority, the BM, promote efficiency significantly more than altruistic punishment. Punishment does not result in increased cooperation above that seen in the BM treatment, but individuals pay sizable costs to punish their counterparts. This causes reduced payoffs and inefficiency. We also find that having a BM from an outgroup witnessing individuals' actions is not beneficial for cooperation. People are clearly sensitive to the presence of a local BM who is active in their village and may thus affect their social image, rather than simply any BM. Our finding complements evidence coming from studies showing that even subtle cues of having one's action being observed enhance cooperation^67^,^68^.

Our results also support the view that, when present, social image concerns ‘crowd out' punishment. We observed that punishment decreases in the presence of the BM. This suggests that any motivation to gain positive reputation by punishing defectors is outweighed by psychological motivations to gain a positive social image with the BM, one who is simultaneously a local authority and a locus for the dissemination of social image information to others in the village. Our results from a naturalistic ‘field' context are in accord with laboratory experiments showing that punishment loses value as a mechanism to build positive social image when it is combined with helping or cooperation^39^,^40^,^41^. For instance, in one study punishment was irrelevant for observers of repeated Public Goods Games in selecting which player to exclude from future play, while cooperative or helping choices were highly relevant^40^. It is only when punishment is a unique observable action that it becomes a relevant factor in positive social image building^50^,^53^,^55^. Moreover, players who had previously performed third party punishment are regarded as more trustworthy in experimental trust games. Nonetheless, when their helping behaviour is also observable, punishment loses its salience as a signal of prosocial disposition^50^. As in our experiments punishment was not alone but was accompanied by a choice of whether to cooperate or defect, it is not surprising that it was not used as a means to build positive social image when the BM was present.

A peculiarity of our experiment is that the presence of the BM, although capable of reducing overall punishment levels compared with the PUN treatment, still leaves a large proportion of players engaging in anti-social punishment. The ultimate cause of such behaviour remains an open question. We argue that in our experiment, spite remains the most probable explanation of anti-social punishment. In many Melanesian societies and in Bougainville in particular, sociability is counterbalanced by an equal measure of competitiveness, which may motivate spiteful behaviour^69^. Moreover, in Teop, which was affected by a civil war up until 2001 (see Supplementary Methods section 1), it is quite possible that people construe social relationships in even more amplified competitive terms. Anti-social behaviours such as reducing others' incomes and increasing one's own status^63^ may thus be seen as strategies directly benefitting the self.

The key finding of our study is that social image concerns outweigh punishment as factors that promote the efficiency of cooperation. This does not mean that punishment has not played a part in the establishment of cooperation in human societies. In fact, a clear structural break has been identified between the manner in which smaller and larger traditional societies enforce pro-social norms of behaviour^52^. According to this classification, Teop, which has fewer than 10,000 inhabitants, belongs to the smaller end of the size spectrum. A plausible hypothesis is that social image concerns prevailed to promote cooperation in early human societies when groups were relatively small and ethnically homogeneous—conditions that still hold today for Teop. This is the case because, we suggest, this type of society is one in which information about social image and reputation can be reliably and efficiently transmitted. As societies grow in size and become more heterogeneous, however, reputational mechanisms may have become less efficient promoters of cooperation (especially before the advent of recent technological innovations such as online rating tools and social media^32^,^33^). This would have necessitated second and third-party punishment, perhaps sequentially, to become important mechanisms in the suite of human behaviours promoting pro-social behaviour^52^,^70^. It is also possible that punishment may play a larger role in promoting cooperation in iterated (rather than one shot) games that allow for learning and the implementation of tit-for-tat strategies^20^,^23^,^25^,^66^. Future studies, examining the roles of social image building, punishment and various combinations of both in one-shot and iterated games, need to be undertaken in societies large and small in order to further illuminate these aspects of the enigma of human cooperation.

## Methods

### Subjects

The study protocol was approved by the Presidential Office of the Institute for the World Economy at Kiel University and the ‘Social and Behavioral Approaches to Global Problems' research area. Approval was also granted by the Regional Government of Bougainville and the Council of Elderly of Teop. Two-hundred and seventy-two participants—143 male and 129 female—provided verbal informed consent and voluntarily participated in 19 experimental sessions across 8 villages (see Supplementary Figs 5 and 6). The sample size was chosen in accordance with standard methods in experimental research on cooperation, aiming to include around 50–60 participants per treatment. As argued in the Supplementary Methods section 2.2, the size of the External BM treatment was lower because of both logistical constraints and because this treatment was mainly meant to be a ‘robustness check' with respect to the BM treatment. Participants belong to the ethno-linguistic group of Teop, one of the 21 ethno-linguistic groups living in the island of Bouganville, an autonomous region of Papua New Guinea. The sample was randomly drawn in each village, under the constraint that at least one person from each household would participate. This ensured a comprehensive level of social stratification in our sample. Each participant only took part in one session and one treatment.

### Experimental procedures

Procedures followed those set out by Henrich *et al*.^17^. Experimental protocol and instructions, as well as additional details on the design and the sampling strategy are reported in the Supplementary Methods sections 2 and 3. Participants were summoned in the ‘waiting area' and were assigned an ID number to guarantee their anonymity. Unlike Henrich *et al*.^17^, the game was never introduced at this stage, to minimize the risk of collusion or contagion. After having offered a general introduction to the procedures and the activities to be carried out during the session, participants provided verbal consent to their participation. Participants were told they could leave the session at any time and for any reason. Subsequently, participants were randomly assigned to one of two experimenters in two separated ‘playing areas' (see Supplementary Fig. 7). The experimenters were fully blinded to the allocation. The game was illustrated using a playing board and real money (see Supplementary Fig. 8). Participants' comprehension was tested asking them to calculate payoffs corresponding to different actions. Only participants who answered correctly four comprehension questions relative to individual payoffs in the PD—and six additional questions in treatments involving punishment - were allowed to take part in the game. In total, we dismissed 8% of the participants for failing the comprehension check.

### Statistical methods

We use logistic linear regressions to analyse Cooperation, ordered logistic linear regression for Punishment and Tobit linear regression for Payoff. This is justified by the dichotomous and discrete nature of Cooperation, the discrete nature of Punishment, and the continuous and censored (at the lower bound of zero and at the upper bound of K34) of Payoff. The regressions include controls for gender, experimenter, comprehension and village effects. All the main results, and particularly those pertaining to payoffs, are robust to either the exclusion of any controls or the inclusion of further demographic controls and robustness checks. The econometric analyses are illustrated in the Supplementary Discussion.

### Data availability

The authors declare that the data supporting the findings of this study are available within the Supplementary Information files of the article. Relevant codes for performing statistical analysies are also provided. Experimental protocols are also included in the Supplementary Information.

## Additional information

**How to cite this article**: Grimalda, G. *et al*. Social image concerns promote cooperation more than altruistic punishment. *Nat. Commun.* 7:12288 doi: 10.1038/ncomms12288 (2016).

## Supplementary Material

Supplementary Information Supplementary Figures 1-8, Supplementary Tables 1-14, Supplementary Discussion, Supplementary Methods and Supplementary References

Supplementary Data 1 The Dataset includes all the variables used in the analysis in both main content and Supplementary Information. Econometric analysis codes for the statistical software Stata are also provided for the analyses included in the main content.

## Figures and Tables

**Table 1:** Experimental design.

**Treatments**	**Observer**	**Punishment**	**Description**	**Observations**
Baseline	Absent	Not available	Standard PD	70
BM	Local BM	Not available	As baseline, with local BM observing participant's choice in the PD. Intended to capture social image concerns	61
BM EXT	External BM	Not available	As baseline, with BM from different ethno-linguistic group observing participant's choice in the PD. Intended to verify social image concerns when the BM's social distance is greater than for local BM	27
PUN	Absent	Available	PD as baseline, followed by punishment stage. Intended to examine effectiveness of punishment option on cooperation	57
BM+PUN	Local BM	Available	As PUN, with local BM observing participant's choices in both the PD and punishment stage. Intended to analyse the interaction of social image concerns with altruistic punishment	57

BM, Big Man; BM EXT, Big Man External; BM+PUN, Big Man+Punishment; PD, prisoner's dilemma; PUN, Punishment.
