# Supplementary material for: Social image concerns promote cooperation more than altruistic punishment
Source: Nat Commun. 2016 Aug 9;7:12288. doi: 10.1038/ncomms12288 (PMC4980489; doi:10.1038/ncomms12288)
Supplement: Supplementary Information — Supplementary Figures 1-8, Supplementary Tables 1-14, Supplementary Discussion, Supplementary Methods and Supplementary References [file ncomms12288-s1.pdf]

Supplementary Figures

Supplementary Figure 1: Comparison of punishment patterns between Teop and Germany

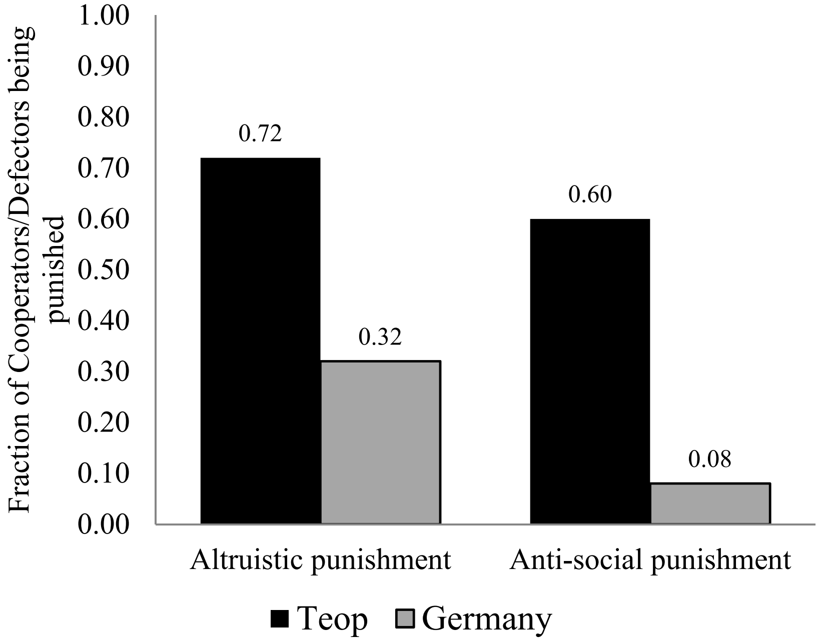

Note: The graph reports the percentage of cooperators and defectors who were punished in our experiment in Teop in the PUN treatment, and in the replication of the PUN treatment that we conducted in Germany.

Supplementary Figure 2: Comparison of punishment patterns in Teop and Russia

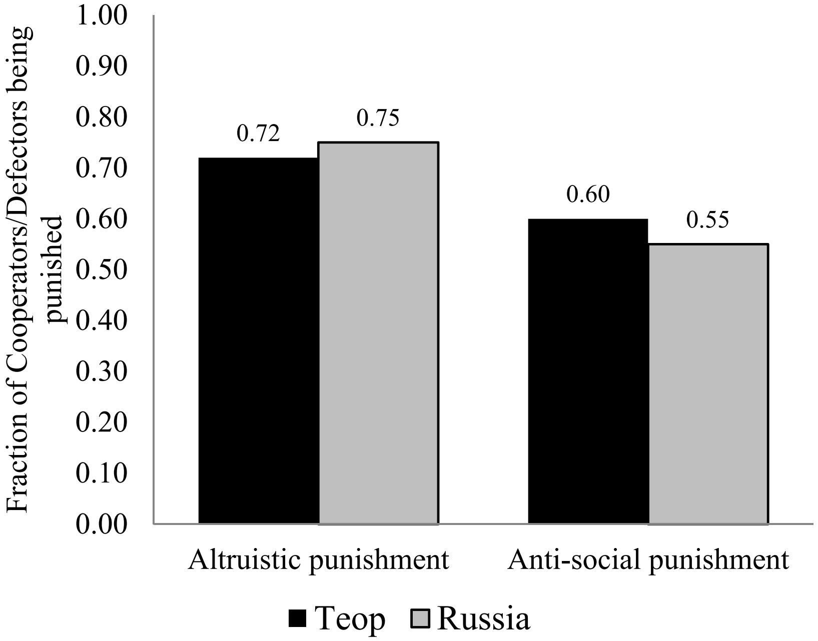

Note: The graph reports the percentage of "high cooperators" and "high defectors" who were punished in the experiment by Gächter and Herrmann (2011) conducted in Russia, and the percentage of co-operators and defectors that were punished in Teop. In Gächter and Herrmann (2011) experiment participants could contribute from 0 to 20 tokens to the collective account. In our experiment participants only had the choice of whether to contribute all of their endowment or nothing. We have classified as anti-social punishment in Gächter and Herrmann (2011) all punishment directed to players who had contributed more than 15 tokens and as altruistic punishment, punishment of those who contributed less than 5 tokens. The graph plots the percentage of punishment for each of these two categories. Very similar percentages would be obtained both if the thresholds to define "high cooperator" and "high defectors" were modified by three tokens, and if high cooperators and high defectors were defined as those whose contribution exceeded or were below the group average by 5 tokens, respectively. (Gächter and Herrmann, 2011, use a measure similar to the latter in their paper to define anti-social and altruistic punishment). In our experiment, anti-social punishment and altruistic punishment is punishment directed to cooperators or defectors, respectively, and the graph plots the percentage of such players who were punished. We consider the average punishment in BM+PUN and PUN. We do not report alternative measures, such as the percentage of the endowment spent on punishment, because these are more difficult to compare. In Gächter and Herrmann (2011) the punishment endowment was not fixed as in our experiment, but depended on the PD payoffs obtained in the previous stage.

Supplementary Figure 3: Patterns of third party punishment in Teop

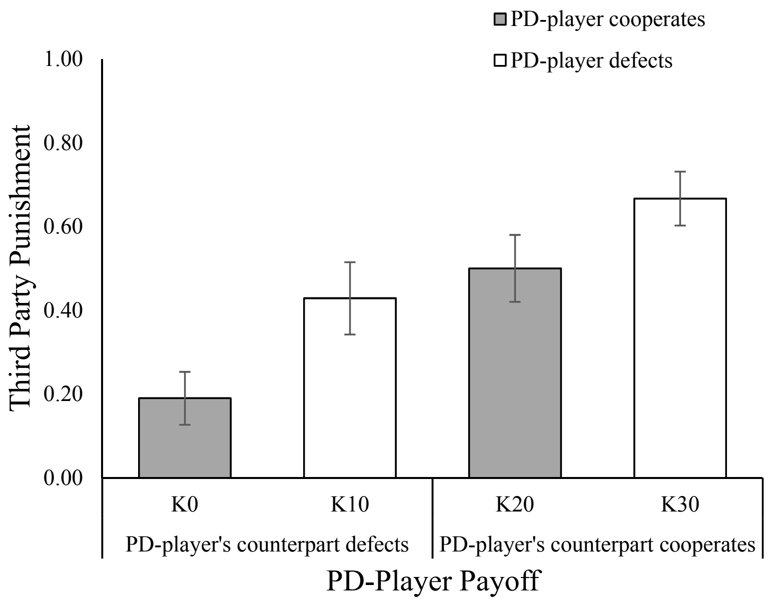

Note: Each of the four columns indicates the average value of the Fraction of the 4K endowment spent on punishment by the third party, for each of the four possible PD outcomes. Third parties were asked to indicate their desired levels of punishment hypothetically for each of the four possible PD outcomes (under the so-called "strategy method" (Brandts and Charness, 2000; Brosig et al., 2003; Oxoby et al., 2004). Outcomes are ordered according to the money in possession of the PD player to whom punishment can be directed. This is represented on the horizontal axis in Kina. Error bars reflect ±1 s.e.m.

Supplementary Figure 4: Expected net payoffs per treatment

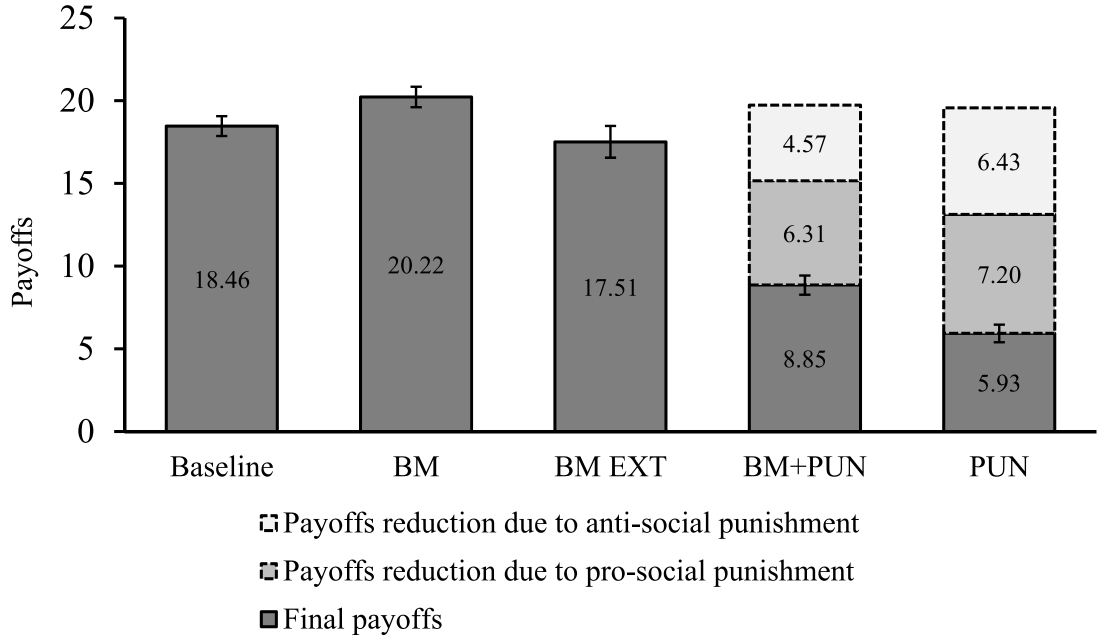

Note: The darkest bars represent earnings net of all punishment costs (payoffs- costs of punishment). The region with intermediate shade represents the earnings that are lost because of costs associated with pro-social punishment – i.e. punishment of defectors. Adding this area to the previous one gives the hypothetical payoffs if anti-social punishment costs were cancelled. The region with lighter shade represents the earnings that are lost because of costs associated with anti-social punishment – i.e. punishment of cooperators. Adding this area to the previous two areas gives the hypothetical payoffs if all punishment costs were cancelled.

Supplementary 5: A coastal village

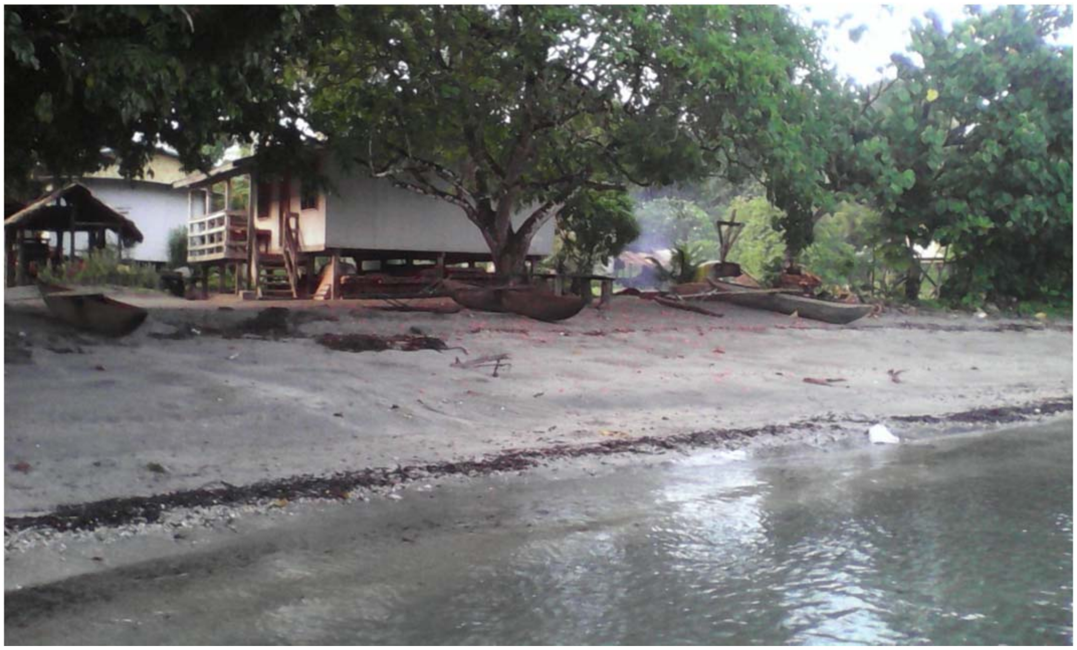

Supplementary Figure 6: A mountain village

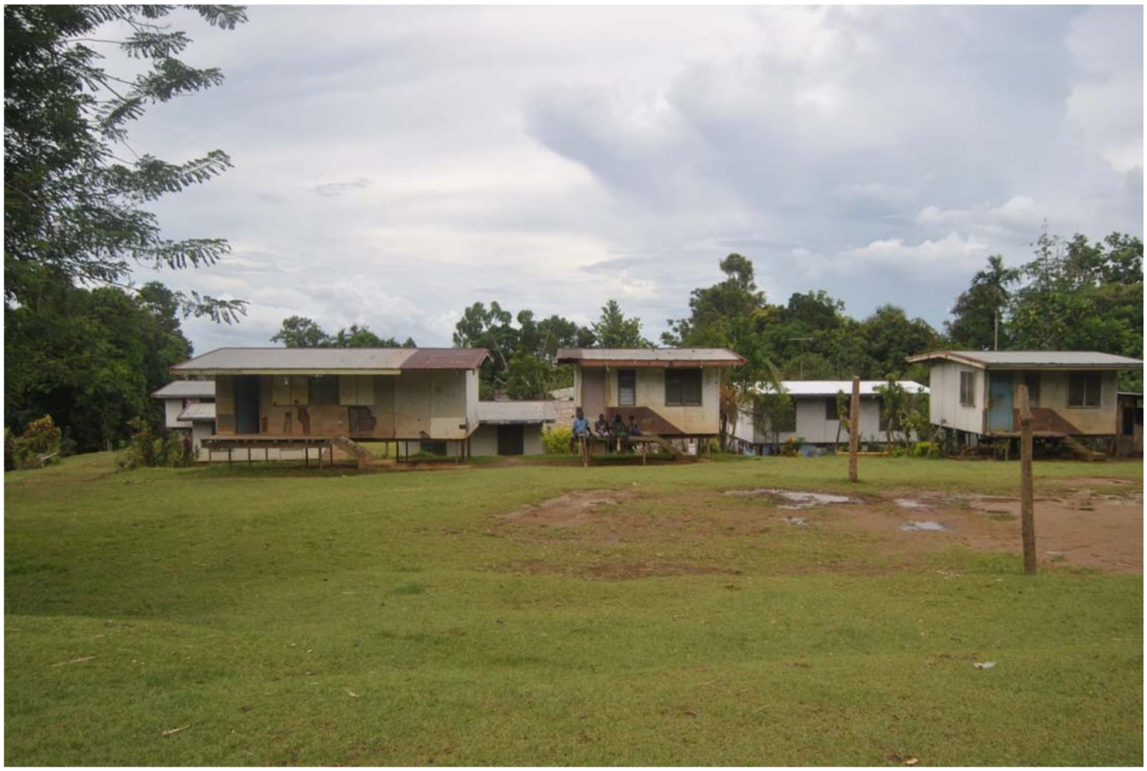

Supplementary Figure 7: A playing area

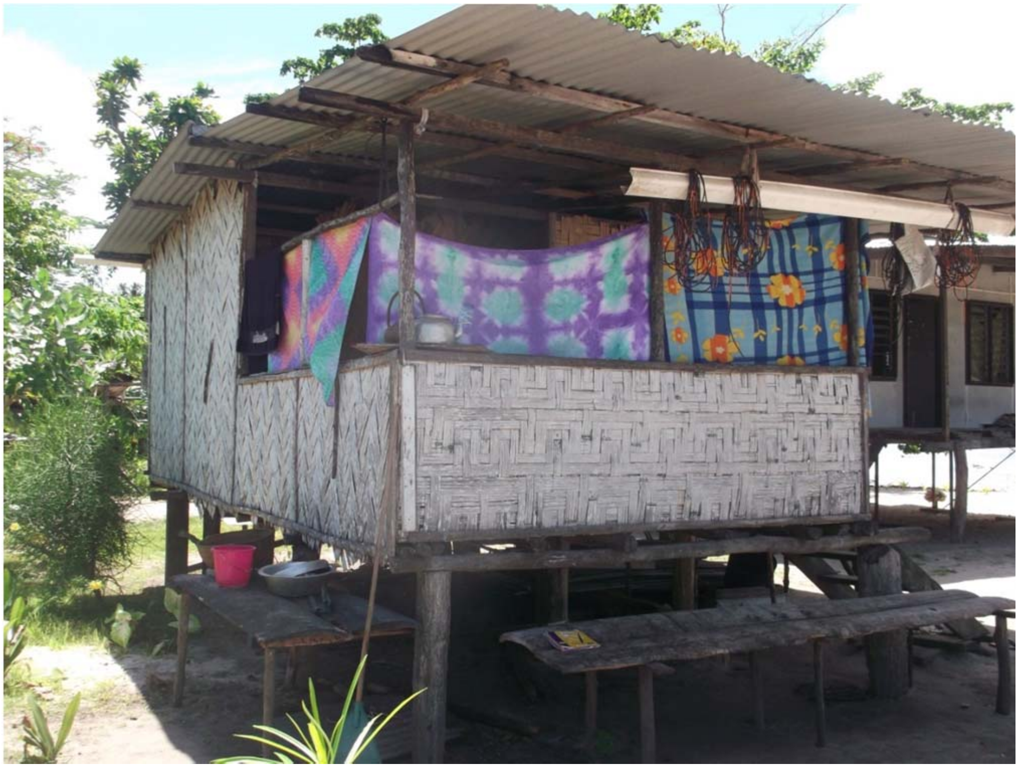

Supplementary Figure 8: The playing board

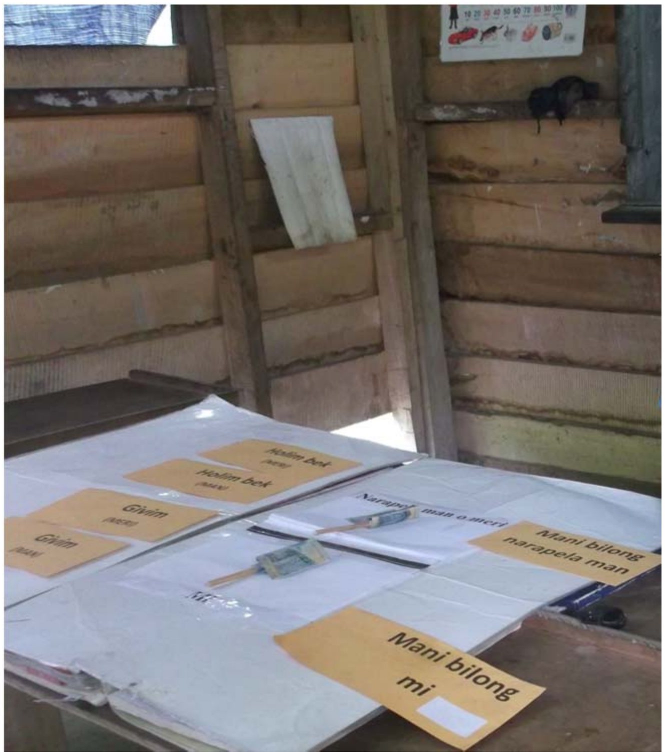

Supplementary Tables

Supplementary Table 1: Descriptive statistics for demographics

Treatment	Variable	Gender (1=Female)	Age	Education	Wealth
ALL	Mean	0.47	36.45	1.01	0.63
	St. Dev.	0.50	12.33	0.41	0.87
	Min	0	17	0	0
	Max	1	77	2	3
	N	272	253	253	272
Baseline	Mean	0.53	35.06	1.05	0.63
	St. Dev.	0.50	13.01	0.41	0.92
	Min	0	17	0	0
	Max	1	77	2	3
	N	70	67	65	70
BM	Mean	0.51	37.98	0.95	0.49
	St. Dev.	0.50	11.81	0.40	0.79
	Min	0	17	0	0
	Max	1	77	2	3
	N	61	56	57	61
BM EXT	Mean	0.44	35.12	1.00	0.63
	St. Dev.	0.51	12.49	0.42	0.74
	Min	0	19	0	0
	Max	1	60	2	2
	N	27	25	24	27
PUN+BM	Mean	0.37	37.04	0.98	0.81
	St. Dev.	0.49	12.61	0.41	0.95
	Min	0	18	0	0
	Max	1	66	2	3
	N	57	52	54	57
PUN	Mean	0.49	36.66	1.06	0.60
	St. Dev.	0.50	11.82	0.41	0.86
	Min	0	17	0	0
	Max	1	56	2	3
	Number of answers	57	53	53	57
K-Wallis test	$\chi^2(4)$	3.82	2.58	2.82	4.09
	P-value	0.43	0.58	0.59	0.39

Note: Education is a categorical variable which takes on the values 0 (below 6 years of education), 1 (6 to 10 years of education), and 2 (above 10 years of education). Wealth is defined as the sum of livestock types owned. We consider the following livestock: pigs, chicken, and rooster. The index takes on the values 0 (holding no livestock types), 1 (holding 1 out of 3 livestock types), 2 (holding 2 out of 3 livestock types), and 3 (holding all three types of livestock). The last line reports the result of a series of Kruskall-Wallis tests on the null hypothesis that the distribution of each of the four demographic characteristics considered is the same across treatments. Such tests never reject the null hypothesis, thus confirming the exogeneity of the treatments with respect to such characteristics. The unbalanced number of answers is due to participants refusing to answer some questions in the questionnaire.

Supplementary Table 2: Cooperation rates and Payoffs by treatment

	Cooperation		Payoff		
Treatment	Mean	Std. Dev.	Mean	Std. Dev.	Obs.
Baseline	0.47	0.50	18.5	5.02	70
BM	0.64	0.48	20.2	4.8	61
BM EXT	0.41	0.50	17.5	5.0	27
BM+PUN	0.61	0.49	8.8	4.4	57
PUN	0.60	0.49	5.9	4.0	57
Total	0.56	0.50	14.1	7.5	272

Note: Cooperation is a dichotomous variable identifying participants who gave their K10 Endowment to the counterpart. Payoff is the final earnings from the game, expressed in Kina, after all the costs associated with punishment have been deducted in treatments BM+PUN and PUN (see SI: Supplementary Discussion, Section 1.6).

Supplementary Table 3: Logit analysis of Cooperation.

Dependent variable	'Cooperation'			
	(1)	(2)	(3)	(4)
Treatments:				
BM	0.69+	0.84*	0.87*	0.89*
	(0.36)	(0.38)	(0.39)	(0.42)
	[0.06]	[0.03]	[0.02]	[0.03]
BM EXT	-0.26	0.19	0.06	0.12
	(0.46)	(0.55)	(0.56)	(0.62)
	[0.57]	[0.73]	[0.91]	[0.84]
PUN	0.58	0.73+	0.69+	0.82+
	(0.36)	(0.39)	(0.39)	(0.45)
	[0.11]	[0.06]	[0.08]	[0.07]
BM+PUN	0.51	0.64+	0.65+	0.59
	(0.36)	(0.38)	(0.38)	(0.42)
Controls:	[0.16]	[0.10]	[0.09]	[0.16]
Gender			-0.24	-0.41
			(0.26)	(0.29)
			[0.36]	[0.15]
Age				0.03*
				(0.01)
				[0.02]
Education (6 to 10 years)				1.25*
				(0.61)
				[0.04]
Education (above 10 years)				1.69*
				(0.78)
				[0.03]
Wealth				0.27
				(0.18)
				[0.14]
Experimenter			-0.3	-0.16
			(0.29)	(0.35)
			[0.30]	[0.65]
Comprehension prisoner's dilemma			0.22	0.29
			(0.16)	(0.19)
			[0.17]	[0.13]
Constant	-0.11	0.08	0.28	-2.63*
	(0.24)	(0.51)	(0.61)	(1.06)
	[0.63]	[0.87]	[0.64]	[0.01]

Supplementary Table 3 (Continued)	(1)	(2)	(3)	(4)
Wald test of equality of coefficients				
BM vs. BM EXT	0.95+	0.65	0.81	0.77
	(0.47)	(0.55)	(0.55)	(0.61)
	[0.05]	[0.24]	[0.14]	[0.21]
BM vs. BM+PUN	0.11	0.11	0.18	0.08
	(0.38)	(0.41)	(0.42)	(0.46)
	[0.77]	[0.79]	[0.67]	[0.87]
BM vs. PUN	0.18	0.20	0.21	0.30
	(0.38)	(0.40)	(0.41)	(0.44)
	[0.63]	[0.62]	[0.60]	[0.50]
BM_EXT vs. BM+PUN	-0.84+	-0.54	-0.63	-0.69
	(0.48)	(0.59)	(0.59)	(0.66)
	[0.08]	[0.36]	[0.29]	[0.30]
BM_EXT vs. PUN	-0.77	-0.45	-0.59	-0.47
	(0.48)	(0.59)	(0.59)	(0.65)
	[0.11]	[0.45]	[0.31]	[0.47]
BM+PUN vs. PUN	0.07	0.09	0.04	0.22
	(0.38)	(0.37)	(0.38)	(0.44)
	[0.85]	[0.82]	[0.92]	[0.61]
Village fixed effects	No	Yes	Yes	Yes
N	272	272	272	243
Pseudo R^2	0.02	0.05	0.06	0.11
Log lik.	-182.99	-177.44	-175.49	-149.74
Percentage of correctly predicted outcomes	59.19 %	59.56 %	63.50 %	65.84 %

Note: A logistic regression model has been fitted. Huber-Whyte heteroschedasticity-robust standard errors are reported in parentheses. Corresponding p-values are reported in square brackets. The dependent variable is Cooperation. It equals 1 if a participant gave the sum of K10 to the counterpart and 0 if they kept it. The Wald tests reported at the bottom of the table are run on the null hypothesis that pairs of dummy coefficients identifying a treatment are equal to each other. Predicted outcomes are computed from the model predicted probability of acceptance by assigning a predicted outcome of acceptance (rejection) whenever the predicted probability is greater (smaller or equal) to 0.5. Thus, a predicted outcome is correct when it matches the actual decision of the subject, i.e. when the subject accepted (rejected) an offer and the model predicted a probability greater (smaller or equal) than 0.5. See Wooldridge (2002).
+ = Statistical significance at the 10 % level; * = Statistical significance at the 5% level; ** = statistical significance at the 1% level; *** = statistical significance at the 0.1% level.

Supplementary Table 4: Logit analysis of Cooperation: further specifications

Dependent variable	'Cooperation'								
	Alternative wealth measures			Individual market integration				Religion	
	(1)	(2)	(3)	(4)	(5)	(6)	(7)	(8)	(9)
Treatments:									
BM	0.87*	0.87*	0.86*	0.89*	0.88*	0.93*	0.87*	0.92*	0.85*
	(0.39)	(0.39)	(0.39)	(0.38)	(0.40)	(0.43)	(0.41)	(0.39)	(0.40)
	[0.03]	[0.02]	[0.03]	[0.02]	[0.03]	[0.03]	[0.03]	[0.02]	[0.03]
BM EXT	0.06	0.06	0.06	0.10	0.19	0.22	0.07	0.15	0.1
	(0.56)	(0.56)	(0.56)	(0.55)	(0.57)	(0.67)	(0.57)	(0.57)	(0.57)
	[0.92]	[0.92]	[0.91]	[0.85]	[0.73]	[0.74]	[0.91]	[0.79]	[0.87]
BM+PUN	0.69+	0.69+	0.68+	0.76+	0.85*	0.80+	0.74+	0.76+	0.81*
	(0.39)	(0.39)	(0.39)	(0.40)	(0.40)	(0.41)	(0.40)	(0.41)	(0.40)
	[0.08]	[0.08]	[0.08]	[0.06]	[0.03]	[0.05]	[0.07]	[0.06]	[0.04]
PUN	0.65+	0.65+	0.63+	0.70+	0.78+	0.55	0.54	0.69+	0.54
	(0.38)	(0.38)	(0.38)	(0.38)	(0.40)	(0.41)	(0.40)	(0.39)	(0.39)
	[0.09]	[0.09]	[0.10]	[0.07]	[0.05]	[0.19]	[0.17]	[0.08]	[0.17]
Controls:									
Gender	-0.24	-0.24	-0.26	-0.32	-0.29	-0.32	-0.33	-0.36	-0.25
	(0.26)	(0.26)	(0.26)	(0.27)	(0.27)	(0.28)	(0.27)	(0.27)	(0.27)
	[0.36]	[0.35]	[0.32]	[0.22]	[0.28]	[0.26]	[0.22]	[0.18]	[0.35]
Agricultural tools	0.00								
	(0.05)								
	[0.93]								
Other general assets		0.00							
		(0.06)							
		[0.96]							
Materials of house (walls & roof)			-0.15						
			(0.18)						
			[0.39]						
Wage labour (1=yes, 0=No)				-0.90					
				(0.55)					
				[0.10]					
Trade (1=yes, 0=no)					-0.33				
					(0.30)				
					[0.28]				

Supplementary Table 4 (Continued)	(1)	(2)	(3)	(4)	(5)	(6)	(7)	(8)	(9)
Visit main capital weekly						-0.15			
						(0.61)			
						[0.80]			
Visit main capital monthly						-0.03			
						(0.63)			
						[0.97]			
Visit local market weekly							0.07		
							(0.43)		
							[0.87]		
Visit local market monthly							0.25		
							(0.38)		
							[0.51]		
Catholic								0.62	
								(0.42)	
								[0.14]	
Religious attendance									0.31
									(0.28)
									[0.26]
Experimenter	-0.30	-0.30	-0.27	-0.35	-0.16	-0.06	-0.19	-0.13	-0.17
	(0.29)	(0.29)	(0.29)	(0.30)	(0.31)	(0.33)	(0.31)	(0.31)	(0.31)
	[0.31]	[0.31]	[0.36]	[0.24]	[0.60]	[0.84]	[0.54]	[0.68]	[0.59]
Comprehension prisoner's dilemma	0.23	0.23	0.21	0.19	0.14	0.21	0.21	0.18	0.20
	(0.16)	(0.16)	(0.16)	(0.17)	(0.17)	(0.18)	(0.17)	(0.17)	(0.17)
	[0.17]	[0.17]	[0.20]	[0.24]	[0.41]	[0.23]	[0.21]	[0.28]	[0.23]
Constant	0.26	0.27	0.37	0.44	0.34	0.47	0.32	-0.05	0.43
	(0.69)	(0.67)	(0.62)	(0.62)	(0.66)	(0.93)	(0.77)	(0.65)	(0.68)
	[0.71]	[0.69]	[0.55]	[0.48]	[0.61]	[0.62]	[0.67]	[0.93]	[0.53]
Village fixed effects	Yes	Yes	Yes	Yes	Yes	Yes	Yes	Yes	Yes
N	272	272	272	272	257	239	257	262	258
Pseudo R^2	0.06	0.06	0.06	0.07	0.06	0.06	0.06	0.06	0.06
Log-lik.	-175.49	-175.49	-175.12	-174.12	-164.93	-153.25	-165.53	-168.02	-165.86
Percentage of correctly predicted outcomes	62.50 %	62.86 %	62.86 %	62.13 %	61.87 %	65.27 %	61.09 %	61.07 %	59.30 %

Notes: See Supplementary Table 3

Supplementary Table 5: Summary statistics for measures of social distance from local and external Big Man

	Local Big Man	External Big Man
1) Recognition of BM	Acquaintance with Big Man (Based on question: "How well do you know [Name of {Local or External Big Man}]?)"	
I know him very well	71 (67 %)	1 (4 %)
I know him well	31 (29 %)	1 (4 %)
I do not know him	4 (4 %)	2 (8 %)
I do not know him at all	0 (0 %)	21 (84 %)
	106 (100 %)	25 (100 %)
	Acceptance of Big Man Guidance (Based on question: "How closely would you follow the Big Man's advice in a specific situation?")	
I would closely follow his advice	63 (59 %)	6 (24 %)
I would follow his advice	39 (37 %)	14 (56 %)
I would not follow his advice	3 (3 %)	1 (4 %)
I would not follow his advice at all	1 (1 %)	4 (16 %)
	106 (100 %)	25 (100 %)
2) Social Connection	Frequency of past encounters (Based on question: "How often have you met [Name of {Local or External Big Man}] in the past year?")	
Never	0 (0 %)	21 (84 %)
Few times a year	15 (14 %)	4 (16 %)
Every month	13 (12 %)	0 (0%)
Every week	21 (20 %)	0 (0%)
Every day	57 (54 %)	0 (0 %)
	106 (100 %)	25 (100 %)
	Frequency of future encounters (Based on question: "How often do you think you will meet [Name of {Local or External Big Man}] in the next year?")	
Never	0 (0 %)	12 (48 %)
Few times a year	21 (20 %)	12 (48 %)
Every month	14 (13 %)	0 (0%)
Every week	26 (25 %)	1 (4 %)
Every day	44 (42 %)	0 (0 %)
	105 (100 %)	25 (100 %)

Note: One participant declined to answer the last question, so the number of observations drops by one for 'Frequency of future encounters'.

Supplementary Table 6: Factor analysis of questionnaire on 'Social distance with Big Man'

Panel A: Principal Component Factor Analysis: Correlations

Factor	Eigenvalue	Difference	Proportion	Cumulative
Factor1	2.34	1.37	0.59	0.59
Factor2	0.98	0.57	0.24	0.83
Factor3	0.41	0.15	0.10	0.93
Factor4	0.27	0.00	0.07	1.00

LR test: independent vs. saturated: $\chi^2(6) = 176.56$ Prob> $\chi^2 = 0.0000$. Observations=130.

Panel B: Principal Component Factor Analysis: Factor loadings (pattern matrix) and unique variances

Variable	Factor 1	Factor 2	Uniqueness
Acquaintance with BM	0.70	0.57	0.19
Acceptance of BM Guidance	0.75	0.46	0.22
Frequency of Past Encounters	0.84	-0.39	0.14
Frequency of Future Encounters	0.77	-0.54	0.12

Panel C: Confirmatory Factor Analysis

	Model 1	Model 2	
VARIABLES	'Social Distance'	'Recognition of BM'	'Social Connection'
Acquaintance with BM	1	1	
	(0)	(0)	
Acceptance of BM Guidance	1.175***	1.203***	
	(0.305)	(0.252)	
Frequency of Past Encounters	2.217***		1
	(0.524)		(0)
Frequency of Future Encounters	1.795***		0.720***
	(0.406)		(0.130)
Observations	130	130	
χ^2 (df)	36.77 (2)	0.048(1)	
LR test of model vs. saturated; Prob > χ^2	<0.001	0.827	
Comparative Fit Index (CFI)	0.8	1	
Root mean squared error of approximation (RMSEA)	0.366	0	
90% CI, lower bound	0.268	0	
90% CI, upper bound	0.474	0.139	
Probability RMSEA <= 0.05	0	0.852	
Standardized root mean squared residual (SRMR)	0.105	0.002	
Coefficient of Determination (CD)	0.88	0.989	

Note: We report the results of a principal component factor analysis (Panels A and B) and of a confirmatory factor analysis (Panel C) conducted on the four items of the questionnaire assessing the social distance between the Big Man and the participant. The unrotated method was used in the Principal Component Factor Analysis.

Five indicators of goodness of fit have been reported to compare Model 1 and Model 2 in the Confirmatory Factor Analysis. Model 1 assumes the existence of a unique latent variable ('Social Distance') influencing the four measurements, while Model 2 assumes two latent variables ('Recognition of BM' and 'Social Connection') with two measurements each. A likelihood ratio test contrasting the fitted model with a saturated model that has no degree of freedoms measures the extent to which the fitted model is capable of reproducing the original matrix of variances and co-variances of the variables in the model. A P-value above 0.1 is generally assumed to represent an acceptable fit. The CFI compares the fitted model with a baseline model that assumes that there is no relationship among the observed variables. A threshold of 0.95 is normally taken to guarantee the good fit of the model. The RMSEA considers how much error the model produces, taking into account the degrees of freedom. This measure penalises models that have unnecessary complexity. Models having RMSEA below 0.05 are normally considered to have a good fit. A confidence interval for the RMSEA, and the probability that RMSEA is below the 0.05 threshold, have also been reported. The SRMR is a measure of how well a model, on average, reproduces each correlation. SRMR below the 0.05 threshold are supposed to have a good fit. The CD is an overall summary of how well the model fits, ranging from 0 to 1. * = Statistical significance at the 5% level; ** = statistical significance at the 1% level; *** = statistical significance at the 0.1% level.

Supplementary Table 7: Regression analysis of determinants of cooperation in relation to social BM social distance

Dependent variable	'Cooperation'							
	(1)	(2)	(3)	(4)	(5)	(6)	(7)	(8)
Treatment:								
BM+PUN	-0.26	-0.15	-0.36	-0.33	-0.4	-0.41	-0.35	-0.32
	(0.41)	(0.40)	(0.43)	(0.41)	(0.43)	(0.42)	(0.43)	(0.41)
	[0.53]	[0.70]	[0.40]	[0.42]	[0.36]	[0.34]	[0.41]	[0.44]
BM post-questionnaire:								
Acquaintance with BM	0.49				0.24	0.35		
	(0.41)				(0.42)	(0.41)		
	[0.23]				[0.57]	[0.39]		
Acceptance of BM guidance		0.12					-0.25	-0.09
		(0.40)					(0.43)	(0.40)
		[0.76]					[0.56]	[0.82]
Frequency of Past Encounters			0.81*		0.72+		0.91*	
			(0.41)		(0.41)		(0.43)	
			[0.05]		[0.08]		[0.03]	
Frequency of Future Encounters				0.97*		0.89*		1.00*
				(0.45)		(0.45)		(0.44)
				[0.03]		[0.05]		[0.02]
Controls:								
Gender	-0.39	-0.36	-0.34	-0.33	-0.35	-0.35	-0.36	-0.35
	(0.39)	(0.39)	(0.40)	(0.39)	(0.40)	(0.39)	(0.40)	(0.40)
	[0.31]	[0.36]	[0.40]	[0.40]	[0.37]	[0.38]	[0.37]	[0.39]
Experimenter	-0.46	-0.49	-0.48	-0.43	-0.46	-0.39	-0.53	-0.45
	(0.41)	(0.42)	(0.41)	(0.41)	(0.41)	(0.42)	(0.42)	(0.43)
	[0.26]	[0.24]	[0.24]	[0.30]	[0.27]	[0.35]	[0.21]	[0.29]
Comprehension prisoner's dilemma	0.08	0.05	0.05	0.01	0.06	0.04	0.03	0.01
	(0.24)	(0.24)	(0.25)	(0.24)	(0.25)	(0.25)	(0.25)	(0.25)
	[0.74]	[0.84]	[0.85]	[0.96]	[0.79]	[0.86]	[0.89]	[0.98]
Mountain	1.96*	2.05**	2.23**	2.13**	2.16**	2.03*	2.29**	2.14**
	(0.78)	(0.78)	(0.77)	(0.81)	(0.78)	(0.82)	(0.77)	(0.82)
	[0.01]	[0.01]	[0.00]	[0.01]	[0.01]	[0.01]	[0.00]	[0.01]
Constant	0.44	0.62	0.4	0.46	0.3	0.29	0.53	0.52
	(0.60)	(0.62)	(0.58)	(0.57)	(0.61)	(0.60)	(0.62)	(0.62)
	[0.47]	[0.32]	[0.49]	[0.41]	[0.62]	[0.63]	[0.40]	[0.40]
Wald test of equality of coefficients								
Acquaintance vs. Past Encounters					-0.48			
					(0.64)			
					[0.45]			
Acquaintance vs. Future Encounters						-0.54		
						(0.60)		

Supplementary Table 7 (continued)	(1)	(2)	(3)	(4)	(5)	(6)	(7)	(8)
						[0.37]		
Acceptance vs. Past Encounters							-1.16+	
							(0.69)	
							[0.10]	
Acceptance vs. Future Encounters								-1.09+
								(0.63)
								[0.08]
N	131	131	131	130	131	130	131	130
Pseudo R^2	0.07	0.07	0.09	0.09	0.09	0.10	0.09	0.09
Log lik.	-81.13	-81.85	-79.85	-78.60	-79.69	-78.25	-79.68	-78.58

Notes: See Supplementary Table 3.

Supplementary Table 8: Descriptive statistics for amounts and fraction of Endowment II (K4) spent for punishment

	Kina spent (S.D.)	Fraction of endowment (S.D.)	N
Total punishment			
BM+PUN	1.79 (1.25)	0.45 (0.31)	57
PUN	2.28 (1.44)	0.57 (0.36)	57
Altruistic punishment			
BM+PUN	1.89 (1.67)	0.47 (0.42)	57
PUN	2.60 (1.60)	0.65 (0.40)	57
Anti-social punishment			
BM+PUN	1.68 (1.55)	0.42 (0.39)	57
PUN	1.96 (1.87)	0.49 (0.47)	57

Supplementary Table 9: Ordered logit analysis of punishment patterns.

	Total Punishment				Altruistic Punishment				Anti-Social Punishment			
	(1)	(2)	(3)	(4)	(5)	(6)	(7)	(8)	(9)	(10)	(11)	(12)
Treatments:												
PUN	0.54+	0.57+	0.59*	0.68*	0.80*	0.82*	0.88*	1.15**	0.27	0.31	0.33	0.31
	(0.28)	(0.29)	(0.29)	(0.31)	(0.35)	(0.37)	(0.38)	(0.43)	(0.36)	(0.38)	(0.40)	(0.45)
	[0.05]	[0.05]	[0.04]	[0.03]	[0.02]	[0.03]	[0.02]	[0.01]	[0.45]	[0.41]	[0.40]	[0.48]
Controls:												
Gender			-0.05	-0.28			-0.17	-0.57			-0.02	-0.20
			(0.29)	(0.33)			(0.39)	(0.47)			(0.40)	(0.43)
			[0.86]	[0.39]			[0.67]	[0.23]			[0.96]	[0.64]
Age				-0.01				-0.02				-0.01
				(0.01)				(0.02)				(0.02)
				[0.30]				[0.33]				[0.49]
Education (6 to 10 years)				-0.29				0.13				-0.65
				(0.48)				(0.64)				(0.72)
				[0.55]				[0.84]				[0.36]
Education (above 10 years)				0.06				0.26				0.10
				(0.75)				(1.16)				(0.96)
				[0.94]				[0.83]				[0.92]
Wealth				0.22				0.31				0.14
				(0.21)				(0.25)				(0.31)

Supplementary Table 9 (continued)	(1)	(2)	(3)	(4)	(5)	(6)	(7)	(8)	(9)	(10)	(11)	(12)
				[0.30]				[0.20]				[0.65]
Experimenter			0.20	0.31			-0.14	-0.10			0.41	0.59
			(0.33)	(0.38)			(0.47)	(0.55)			(0.43)	(0.50)
			[0.55]	[0.43]			[0.77]	[0.85]			[0.34]	[0.24]
Comprehension punishment stage			0.15	0.20			0.22	0.28			0.10	0.13
			(0.21)	(0.24)			(0.28)	(0.34)			(0.27)	(0.29)
			[0.47]	[0.40]			[0.44]	[0.41]			[0.71]	[0.65]
Comprehension prisoner's dilemma			0.25	0.33			0.33	0.65			0.20	0.14
			(0.21)	(0.28)			(0.32)	(0.44)			(0.27)	(0.35)
			[0.23]	[0.23]			[0.31]	[0.14]			[0.46]	[0.69]
Cut1												
Constant	-0.37+	0.22	0.57	0.53	-0.53*	-0.49	-0.37	-0.08	-0.23	0.88	1.35	1.37
	(0.19)	(0.83)	(0.85)	(1.27)	(0.26)	(1.00)	(0.99)	(1.51)	(0.22)	(1.00)	(1.10)	(2.13)
	[0.05]	[0.79]	[0.50]	[0.68]	[0.04]	[0.63]	[0.71]	[0.96]	[0.29]	[0.38]	[0.22]	[0.52]
Cut2												
Constant	0.81***	1.41+	1.79*	1.79	0.77**	0.82	0.97	1.37	0.86***	2.07*	2.56*	2.6
	(0.18)	(0.85)	(0.85)	(1.29)	(0.27)	(1.00)	(0.99)	(1.53)	(0.22)	(1.02)	(1.11)	(2.16)
	[0.00]	[0.10]	[0.04]	[0.17]	[0.00]	[0.41]	[0.33]	[0.37]	[0.00]	[0.04]	[0.02]	[0.23]
Village fixed effects	No	Yes	Yes	Yes	No	Yes	Yes	Yes	No	Yes	Yes	Yes
N	228	228	228	202	114	114	114	101	114	114	114	101
Log lik.	-246.52	-243.96	-241.55	-210.86	-121.09	-120.51	-118.78	-102.54	-123.03	-117.53	-116.43	-100.35

Note: An ordered logit model has been fitted. Heteroskedasticity-robust clustered standard errors are reported in parentheses for models in columns 1 and 4. Clusters are given by individuals. In models 5-12 we use Huber-White heteroschedasticity-robust standard errors (see SI: Supplementary Discussion, Section 1.1). The corresponding p-values are

reported in square brackets. The dependent variable is Punishment, which takes on the values K0, K2 and K4. The columns report results for the following decisions: Overall Pun.: pooled data of punishment decisions (against both defectors and cooperators); Pro-Social Pun.: data restricted to punishment of defectors; Anti-Social Pun.: data restricted to punishment of cooperators. The results reported in the article are based on the models in column 3, 7 and 11. + = Statistical significance at the 10 % level; * = Statistical significance at the 5% level; ** = Statistical significance at the 1% level; *** = Statistical significance at the 0.1% level.

Supplementary Table 10: Ordered logit analysis of punishment patterns and religion

	Total Pun.		Altruistic Pun.		Anti-social Pun.	
	(1)	(2)	(3)	(4)	(5)	(6)
Treatments:						
PUN	0.51+	0.47+	0.74+	0.77*	0.27	0.18
	(0.31)	(0.28)	(0.41)	(0.39)	(0.41)	(0.40)
	[0.10]	[0.09]	[0.08]	[0.05]	[0.50]	[0.66]
Controls:						
Gender	-0.07	-0.13	-0.11	-0.21	-0.12	-0.15
	(0.32)	(0.29)	(0.42)	(0.42)	(0.42)	(0.42)
	[0.81]	[0.66]	[0.79]	[0.61]	[0.77]	[0.72]
Catholic	-0.25		-0.57		0.04	
	(0.51)		(0.61)		(0.65)	
	[0.62]		[0.35]		[0.95]	
Religious attendance		0.97**		0.93*		1.13*
		(0.31)		(0.42)		(0.44)
		[0.00]		[0.03]		[0.01]
Experimenter	0.33	0.42	-0.01	0.04	0.60	0.73+
	(0.34)	(0.33)	(0.47)	(0.48)	(0.44)	(0.44)
	[0.33]	[0.21]	[0.99]	[0.93]	[0.17]	[0.10]
Comprehension punishment stage	0.17	0.25	0.26	0.31	0.12	0.23
	(0.21)	(0.22)	(0.28)	(0.30)	(0.27)	(0.29)
	[0.41]	[0.25]	[0.36]	[0.30]	[0.66]	[0.42]
Comprehension prisoner's dilemma	0.26	0.04	0.30	0.10	0.22	-0.01
	(0.22)	(0.19)	(0.33)	(0.31)	(0.29)	(0.29)
	[0.25]	[0.83]	[0.36]	[0.74]	[0.45]	[0.97]
Cut1						
Constant	1.09	1.90*	0.12	0.62	2.16	15.40***
	(0.93)	(0.78)	(1.03)	(1.08)	(1.42)	(0.79)
	[0.24]	[0.01]	[0.91]	[0.57]	[0.13]	[0.00]
Cut2						
Constant	2.29*	3.19***	1.46	2.04+	3.33*	16.69***
	(0.95)	(0.80)	(1.03)	(1.10)	(1.45)	(0.81)
	[0.02]	[0.00]	[0.15]	[0.06]	[0.02]	[0.00]
Village effects	Yes	Yes	Yes	Yes	Yes	Yes
N	222	218	111	109	111	109
Pseudo R^2	0.03	0.06	0.04	0.07	0.06	0.11
Log lik.	-233.87	-222.79	-115.35	-110.85	-112.19	-104.28

Note: see Supplementary Table 9.

Supplementary Table 11: Mean fractions of Endowment II (K4) spent by the third-party (S.D.)

Punished person is a	punished person's PD partner cooperates	punished person's PD partner defects
Defector	0.67<br>(0.29)<br>N = 21	0.42<br>(0.40)<br>N = 21
Cooperator	0.50<br>(0.35)<br>N = 21	0.19<br>(0.29)<br>N = 21

Supplementary Table 12: OLS analysis of Third-party punishment: Analysis of PD outcomes effect

Dependent variable	'Third-Party Punishment'		
	(1)	(2)	(3)
D/D	0.24**	0.24*	0.24*
	(0.08)	(0.08)	(0.09)
	[0.01]	[0.01]	[0.01]
C/C	0.31**	0.31**	0.31**
	(0.10)	(0.10)	(0.11)
	[0.01]	[0.01]	[0.01]
D/C	0.48***	0.48***	0.48***
	(0.10)	(0.10)	(0.10)
	[0.00]	[0.00]	[0.00]
Gender			0.15
			(0.11)
			[0.17]
Comprehension punishment stage			0.07+
			(0.04)
			[0.07]
Comprehension prisoner's dilemma			0.04
			(0.05)
			[0.43]
Constant	0.19**	0.20*	-0.02
	(0.07)	(0.09)	(0.12)
	[0.01]	[0.04]	[0.89]
Wald test if coefficients are zero			
H0: D/D = 0	0.43***	0.44***	0.22
	(0.09)	(0.11)	(0.11)
	[0.00]	[0.00]	[0.13]
H0: C/C = 0	0.50***	0.51***	0.29+
	(0.08)	(0.10)	(0.14)
	[0.00]	[0.00]	[0.06]
H0: D/C = 0	0.66***	0.68***	0.46***
	(0.06)	(0.08)	(0.13)
	[0.00]	[0.00]	[0.00]

Supplementary Table 12 (continued)	(1)	(2)	(3)
Wald-test if coefficients of interest are equal to each other			
F-statistic	9.46	9.11	8.75
	[0.00]	[0.00]	[0.00]
Village fixed effects	No	Yes	Yes
N	84	84	84
R-sq.	0.21	0.22	0.28

Note: An ordinary least square model has been fitted. Heteroskedasticity-robust clustered standard errors are reported in parentheses. Clusters are given by individuals. The dependent variable is Punishment, which takes on the values K0, K2 and K4. The columns report results for overall punishment, that is, pooled data of punishment decisions (all four situations that can emerge in the PD).
D/D = The player to whom punishment can be directed defected; her PD partner also defected;
D/C = The player to whom punishment can be directed defected; her PD partner cooperated;
C/C = The player to whom punishment can be directed cooperated; her PD partner also cooperated;
C/D = The player to whom punishment can be directed cooperated; her PD partner defected (reference group).
The Wald tests reported at the bottom of the table are run on the null hypothesis that 1) each coefficient of interest is equal to zero and 2) all coefficients of interest are equal to each other. + = Statistical significance at the 10 % level; * = Statistical significance at the 5% level; ** = Statistical significance at the 1% level; *** = Statistical significance at the 0.1% level.

Supplementary Table 13: OLS analysis of Third-party punishment: Impact of PD-player payoff

Dependent variable	'Third-Party Punishment'		
	(1)	(2)	(3)
PD-player payoff	0.15***	0.15***	0.15***
	(0.03)	(0.03)	(0.04)
	[0.00]	[0.00]	[0.00]
Gender			0.15
			(0.11)
			[0.16]
Comprehension punishment stage			0.07+
			(0.04)
			[0.07]
Comprehension prisoner's dilemma			0.04
			(0.05)
			[0.42]
Constant	0.22**	0.23*	0.01
	(0.07)	(0.10)	(0.13)
	[0.01]	[0.02]	[0.91]
Village fixed effects	No	Yes	Yes
N	84	84	84
R-sq.	0.21	0.21	0.27

Note: An ordinary least square model has been fitted. Heteroskedasticity-robust clustered standard errors are reported in parentheses. Clusters are given by individuals. The dependent variable is Punishment, which takes on the values K0, K2 and K4.
+ = Statistical significance at the 10 % level; * = Statistical significance at the 5% level; ** = Statistical significance at the 1% level; *** = Statistical significance at the 0.1% level.

Supplementary Table 14: Tobit regression models for individual payoffs

	Payoff				Payoff if Anti-Social Punishment Costs=0				Payoff if All Punishment Costs=0			
	(1)	(2)	(3)	(4)	(5)	(6)	(7)	(8)	(9)	(10)	(11)	(12)
Treatments:												
BM	2.71*	2.54*	2.51*	2.59*	2.48*	2.30*	2.29*	2.27*	2.80**	2.54*	2.50*	2.62*
	(1.05)	(1.03)	(1.03)	(1.06)	(1.01)	(0.98)	(0.98)	(1.02)	(1.07)	(1.05)	(1.05)	(1.08)
	[0.01]	[0.01]	[0.02]	[0.02]	[0.02]	[0.02]	[0.02]	[0.03]	[0.01]	[0.02]	[0.02]	[0.02]
BM EXT	-0.95	-1.96	-1.67	-1.88	-0.95	-1.82	-1.74	-1.95	-2.18	-3.39*	-3.10+	-3.14+
	(1.12)	(1.36)	(1.35)	(1.41)	(1.12)	(1.30)	(1.29)	(1.33)	(1.50)	(1.68)	(1.66)	(1.72)
	[0.40]	[0.15]	[0.22]	[0.18]	[0.40]	[0.16]	[0.18]	[0.14]	[0.15]	[0.04]	[0.06]	[0.07]
BM+PUN	-9.61***	-9.97***	-9.88***	-9.95***	-3.30***	-3.39***	-3.35***	-3.30***	1.26	1.00	1.12	1.02
	(0.83)	(0.86)	(0.86)	(0.91)	(0.60)	(0.65)	(0.66)	(0.74)	(0.88)	(0.91)	(0.91)	(0.98)
	[0.00]	[0.00]	[0.00]	[0.00]	[0.00]	[0.00]	[0.00]	[0.00]	[0.15]	[0.27]	[0.22]	[0.29]
PUN	-13.10***	-13.42***	-13.45***	-13.28***	-5.87***	-5.92***	-5.92***	-6.23***	1.10	0.88	0.87	1.07
	(0.89)	(0.93)	(0.92)	(0.97)	(0.70)	(0.72)	(0.73)	(0.77)	(0.88)	(0.90)	(0.89)	(0.95)
	[0.00]	[0.00]	[0.00]	[0.00]	[0.00]	[0.00]	[0.00]	[0.00]	[0.22]	[0.33]	[0.33]	[0.26]
Controls:												
Gender			0.54	0.96			0.22	0.38			0.71	1.14
			(0.63)	(0.65)			(0.53)	(0.56)			(0.67)	(0.70)
			[0.39]	[0.15]			[0.69]	[0.50]			[0.29]	[0.10]
Age				-0.05*				-0.02				-0.07*
				(0.02)				(0.02)				(0.03)
				[0.03]				[0.38]				[0.02]
Education (6 to 10 years)				-3.32*				-2.22+				-3.70*
				(1.45)				(1.27)				(1.60)

Supplementary Table 14 (Continued)	(1)	(2)	(3)	(4)	(5)	(6)	(7)	(8)	(9)	(10)	(11)	(12)
				[0.02]				[0.08]				[0.02]
Education (above 10 years)				-4.30*				-3.69*				-4.69*
				(1.76)				(1.60)				(1.93)
				[0.01]				[0.02]				[0.02]
Wealth				-0.76+				-0.79*				-0.75+
				(0.41)				(0.34)				(0.42)
				[0.06]				[0.02]				[0.08]
Experimenter			0.56	0.03			0.07	-0.52			0.88	0.34
			(0.73)	(0.82)			(0.61)	(0.67)			(0.76)	(0.85)
			[0.44]	[0.97]			[0.91]	[0.44]			[0.25]	[0.69]
Comprehension prisoner's dilemma			-0.59	-0.73+			-0.15	-0.34			-0.51	-0.65
			(0.40)	(0.43)			(0.33)	(0.37)			(0.42)	(0.45)
			[0.14]	[0.09]			[0.64]	[0.36]			[0.22]	[0.15]
Constant	18.46***	17.87***	17.62***	24.45***	18.46***	17.22***	17.18***	21.69***	18.46***	17.63***	16.90***	24.67***
	(0.60)	(1.15)	(1.43)	(2.33)	(0.60)	(1.07)	(1.27)	(2.09)	(0.60)	(1.22)	(1.50)	(2.49)
sigma	[0.00]	[0.00]	[0.00]	[0.00]	[0.00]	[0.00]	[0.00]	[0.00]	[0.00]	[0.00]	[0.00]	[0.00]
Constant	5.13***	5.03***	4.99***	4.84***	4.27***	4.20***	4.20***	4.12***	5.46***	5.35***	5.31***	5.16***
	(0.15)	(0.15)	(0.16)	(0.17)	(0.16)	(0.16)	(0.16)	(0.17)	(0.15)	(0.15)	(0.15)	(0.17)
	[0.00]	[0.00]	[0.00]	[0.00]	[0.00]	[0.00]	[0.00]	[0.00]	[0.00]	[0.00]	[0.00]	[0.00]
Wald test of equality of coefficients												
BM vs. BM_EXT	3.66**	4.51**	4.18**	4.47**	3.43**	4.11**	4.03**	4.22**	4.99**	5.93**	5.60**	5.76**
	(1.29)	(1.53)	(1.50)	(1.57)	(1.25)	(1.44)	(1.43)	(1.49)	(1.67)	(1.86)	(1.82)	(1.89)
	[0.00]	[0.00]	[0.01]	[0.00]	[0.01]	[0.00]	[0.01]	[0.01]	[0.00]	[0.00]	[0.00]	[0.00]

Supplementary Table 14 (continued)	(1)	(2)	(3)	(4)	(5)	(6)	(7)	(8)	(9)	(10)	(11)	(12)
BM vs. BM+PUN	12.32***	12.52***	12.38***	12.54***	5.78***	5.68***	5.64***	5.56***	1.54	1.53	1.38	1.6
	(1.04)	(1.07)	(1.06)	(1.09)	(0.82)	(0.84)	(0.83)	(0.89)	(1.09)	(1.12)	(1.11)	(1.15)
	[0.00]	[0.00]	[0.00]	[0.00]	[0.00]	[0.00]	[0.00]	[0.00]	[0.16]	[0.17]	[0.21]	[0.17]
BM vs. PUN	15.81***	15.97***	15.95***	15.87***	8.35***	8.22***	8.21***	8.50***	1.70	1.65	1.63	1.55
	(1.10)	(1.15)	(1.15)	(1.18)	(0.92)	(0.90)	(0.90)	(0.93)	(1.10)	(1.11)	(1.09)	(1.13)
	[0.00]	[0.00]	[0.00]	[0.00]	[0.00]	[0.00]	[0.00]	[0.00]	[0.12]	[0.14]	[0.14]	[0.17]
BM_EXT vs. BM+PUN	8.66***	8.01***	8.21***	8.06***	2.35*	1.57	1.61	1.35	-3.45*	-4.40*	-4.22*	-4.16*
	(1.11)	(1.46)	(1.43)	(1.51)	(0.95)	(1.25)	(1.24)	(1.34)	(1.52)	(1.79)	(1.75)	(1.84)
	[0.00]	[0.00]	[0.00]	[0.00]	[0.01]	[0.21]	[0.20]	[0.32]	[0.02]	[0.01]	[0.02]	[0.02]
BM_EXT vs. PUN	12.15***	11.46***	11.78***	11.40***	4.92***	4.10**	4.18**	4.28**	-3.28*	-4.28*	-3.97*	-4.20*
	(1.15)	(1.52)	(1.49)	(1.58)	(1.01)	(1.29)	(1.28)	(1.34)	(1.53)	(1.78)	(1.74)	(1.83)
	[0.00]	[0.00]	[0.00]	[0.00]	[0.00]	[0.00]	[0.00]	[0.00]	[0.03]	[0.02]	[0.02]	[0.02]
PUN vs. BM+PUN	-3.49***	-3.45***	-3.57***	-3.34***	-2.57***	-2.53***	-2.57***	-2.93***	-0.16	-0.12	-0.25	0.04
	(0.88)	(0.86)	(0.84)	(0.89)	(0.38)	(0.40)	(0.41)	(0.48)	(0.92)	(0.90)	(0.88)	(0.96)
	[0.00]	[0.00]	[0.00]	[0.00]	[0.00]	[0.00]	[0.00]	[0.00]	[0.86]	[0.89]	[0.78]	[0.96]
Village fixed effects	No	Yes	Yes	Yes	No	Yes	Yes	Yes	No	Yes	Yes	Yes
N	0.14	0.14	0.15	0.16	0.07	0.07	0.08	0.09	0.01	0.02	0.02	0.03
Pseudo R^2	-764.91	-759.63	-757.83	-665.98	-721.74	-717.33	-717.14	-632.62	-779.75	-774.45	-772.43	-681.26
Log lik.	272	272	272	243	272	272	272	243	272	272	272	243

Note: A Tobit model has been fitted. Huber-White heteroskedasticity-robust standard errors are reported in parentheses. The corresponding p-values are reported in square brackets. The dependent variable is Individual Expected payoff. This is computed as the payoff resulting from an individual playing the actions he or she performed in our experiment, combined with the average of the decision taken by participants in a certain treatment. In columns 1 to 4 expected payoffs are calculated by subtracting all punishment costs. These are therefore the final payoffs that people took home. The results reported in the article are based on the model in column 3. In columns 5 to 8 expected payoffs are calculated under the hypothetical condition that costs of anti-social punishment are equal to zero. In columns 9 to 12 expected payoffs are calculated under the hypothetical condition that all costs of punishment are equal to zero. + = Statistical significance at the 10% level, * = statistical significance at the 5% level; ** = statistical significance at the 1% level; *** = statistical significance at the 0.1% level.

Supplementary Discussion

1.1 Introduction to econometric analysis

We used three statistical methods for the econometric analysis in the present study. All analyses and P-values reported in the main text are taken from the following regression models:

Logit model:

The variable "Cooperation" equals 1 if a participant gave the sum of K10 to the counterpart and 0 if they kept it. Due to the dichotomous nature of this variable, we fit a logistic linear regression model (logit model). A logit model predicts a binary response by maximum likelihood. It models the probability of a positive outcome given a set of regressors. In all logit regression models we use Huber-White standard-errors robust to heterosckedasticity (Huber, 1967; White, 1980).

Ordered logit model:

Punishment is an ordinal variable and takes on the values K0, K2 and K4. Given the discrete nature of this variable, and the fact that possible outcomes are ordered and greater than two, we run an ordered logistic linear regression model. In regressions where both punishment of defectors and cooperators are included (see Supplementary Table 9, columns 1 and 2) we have two observations for each individual - one for desired punishment when the other person defects, and one for desired punishment when the other person gives. In this case we use heteroskedasticity-robust standard errors clustered at the level of the individual (Kish and Frankel, 1974; Cameron and Trivedi, 2005). This method models observations coming from the same individual as being correlated, and observations coming from different individuals as being uncorrelated. This method is appropriate only as the number of clusters goes to infinity. With 57 clusters in our estimates, we believe that this requirement can be considered to be satisfied. When the number of observations within each cluster is equal to one, this method trivially coincides with the heteroskedasticity- robust Huber-White standard errors (Cameron and Trivedi, 2005). In other regressions we only use observations for punishment of either defectors (Supplementary Table 9, columns 3-4) or cooperators (Supplementary Table 9, columns 5-6), and we therefore revert to the use of Huber-White standard errors.

Tobit model:

The Tobit model, also called a censored regression model, is designed to estimate linear relationships between variables when there is either left- or right-censoring in the dependent variable (also known as censoring from below and above, respectively) and the dependent

variable is continuous. In our case, payoffs are defined on the interval ranging from zero (due to rationing rules, a participant could never earn below zero) and 34 (the highest amount possible), so a Tobit model is appropriate. In all all Tobit regression models we use Huber-White standard-errors robust to heteroskedasticity.

1.2 Analysis of cooperation rates

Regression Analysis:

Here we report the full econometric models for the regressions presented in the paper regarding cooperation. The model reported in Supplementary Table 3: column 1 includes only treatment effects. In column 2 we add village fixed effects as controls. Column 3 further adds comprehension and experimenter identity effect as controls. The comprehension variable is derived from the number of mistakes participants made when answering our examples. It has a discrete form and is coded in the following way: (0) no mistakes, (1) one mistake and (2) more than one mistakes. 'Experimenter' is a dummy variable identifying one of the two researchers conducting the experiment. This is the regression from which the tests reported in the article, Figure 1, are drawn. The model in the fourth column adds further demographic controls – namely, age and educational attainment - whose introduction comes at the cost of losing observations due to non-responses in the questionnaire. For this reason we report both models with and without extra demographic controls.

Supplementary Table 4 presents logit regressions with alternative specifications to those included in Supplementary Table 3. Columns 1 to 3 include models with different wealth measures – specifically, the number of agricultural tools owned by the responder -variable 'Agricultural tools' – the number and type of other general assets owned by the participant, such as water tanks,chairs, mattresses, electricity generators – variable 'Other general asset'-, and the material which the roof and walls of a house are built, – variable 'Materials of house'. The latter variable takes on the value 0 if both roof and walls are made with commercial materials (e.g., processed wood, concrete, sheet), 1 if either the roof or the wall is made out of traditional bush materials (e.g., sago or mangrove wood) and 2 if both -roof and walls - are made out of traditional bush materials. Columns 4 to 7 include variables controlling for individual market integration, i.e. whether the individual earns a wage, or performs some kind of trade or sales of her own produces, and how frequently she visits the main capital of the region or the local markets. Columns 8 and 9 include variables controlling for religion affiliation. The variable 'Catholic' identifies whether the individual declares her religion affiliation to be Catholic as opposed to one the several Protestant denominations being present in Teop, e.g. United Church or Methodist. Around 23% of the respondents are Catholic. Interestingly, all participants answered that they had some religious affiliation. The variable 'Religious attendance' is a dummy variable identifying participants with lower than the median attendance of religious services, i.e. those attending less frequently than at least

once a week. Note that observations in Supplementary Table 4 are not balanced due to missing values in the market integration and religion variables.

The main result – Cooperation is highest in the BM treatment compared to baseline – is robust troughout all models. Apart from age and educational attainment, who covaries positively with cooperation (see main paper, 'Results' section), none of these alternative wealth measures, market integration and frequency of religion attendances are significant predictors of cooperation. Being active in trade exerts a negative effect on cooperation, which is, however, statistically not significant (P = 0.103, N = 272; Supplementary Table 4, column 5). Declaring to be Catholic is positively associated with cooperation, and again the effect is statistically not significant (P = 0.136, N = 262; Supplementary Table 4, column 8).

We also find significant differences in the level of cooperation between groups of villages. Villages can be grouped into a mountain (village 1 and 7) or a coastal environment (remaining villages; see Supplementary Fig. 5 and 6). A Wald test on the null hypothesis that village dummy coefficients are all equal to each other shows that there are no differences among either mountain villages (P = 0.634) or costal villages (P = 0.192). However, the null hypothesis that all villages coefficients are equal to each other is rejected (P = 0.034). This implies that a statistically significant difference in cooperation rates exists between the two environments, with mountain villages being more cooperative than coastal villages. The same difference remains adding the four villages where we ran the follow-up study (see SI: Supplementary Discussion, Section 2.1), one of which belongs to the mountain and three to the coastal environment. Since the main road of the island passes near the coast and in most cases through villages, coastal villages are more easily interconnected among themselves and also with Buka, the main market town of the Bougainville archipelagos. Mountain villages are more sparsely distributed and isolated from other villages. Walking to the main road can take from one hour to three hours. In case of heavy rain, pick-up trucks cannot travel on roads connecting to the main road. We can speculate that the difference we observe in our analysis may be due to norms of cooperation being more strongly upheld in mountain villages precisely because of their higher isolation.

1.3 Analysis of social distance from Big Man

We obtained some measures of the social distance between participants and Big Men through a post-experiment questionnaire. This was only run in treatments where the BM had been present. The text of the questions is reported in Supplementary Table 5. The variable that we call 'Acquaintance with BM' measures how well a participant knows the Big Man. The variable 'Acceptance of BM Guidance' asks how closely an individual would follow the Big

Man's advice in a specific situation. Four possible answers ranged from "I would closely follow his advice" to "I would not follow his advice at all". We expect that the higher the social standing or the respect that a Big Man can command, the higher individuals' acceptance of BM guidance. The variables that we call 'Frequency of Past Encounters' and 'Frequency of Future Encounters' inquired about the frequency of encounters between the BM and the participant in the past year and the predicted frequency in the next year.

Firstly, we note that responses regarding the local Big Man were largely the same regardless of whether a subject participated in treatment BM or treatment BM+PUN. A Wilcoxon Mann-Whitney test fails to reject the null hypothesis that the observations have been generated by the same distribution for all four questions ($P = 0.65$, $N = 106$ for 'Acquaintance with BM'; $P = 0.81$, $N = 106$ for 'Acceptance of BM Guidance'; $P = 0.96$, $N = 106$ for 'Frequency of Past Encounters'; $P = 0.60$, $N = 105$ for 'Frequency of Future Encounters'; All tests are two-tailed). In the ensuing analysis we thus conflate the observations from BM and BM+PUN, and contrast the results for the local vis-à-vis the external Big Man.

Participants clearly perceived the external Big Man as more socially distant than the local BM under all of the above accounts (See Supplementary Table 5). 59% of participants reported that they would follow the local Big Man's advice closely, while the same was true for only 24% of the participants in the case of the external Big Man. 74% of participants reported that in the past year they had met the local Big Man at least once every week, while none reported this for the external Big Man. Mann-Whitney tests reject in all of four cases that the distribution of responses in BM EXT is generated by the same distribution as in BM and BM+PUN ($P<0.001$). The same result would be obtained if we compared BM EXT with either BM or BM+PUN separately (not reported, available upon request). These summary statistics show that the local Big Man is inextricably connected to the social network and has higher authority compared to his external counterpart. Thus, we conclude that the treatments BM and BM EXT are appropriate instruments to examine and verify social image concerns.

We hypothesise that these variables reflect the social distance that the participant experiences vis-à-vis the Big Man. We also hypothesise that this construct comprises two separate sub-dimensions. The first two questions ('Acquaintance with BM' and 'Acceptance of BM Guidance') measure what we call the 'Recognition of BM', because they relate to the self-reported participant's degree of familiarity with the Big Man and the extent to which the Big Man is recognised as offering legitimate guidance in every day's affairs. The other two variables ('Frequency of Past Encounters' and 'Frequency of Future Encounters') clearly

reflect the degree to which the participant and the Big Man are connected in the social network.

We now substantiate this claim through factor analysis. Since in the ensuing econometric analysis we use these variables in a dichotomous format, we carry out the factor analysis on the dummy variables that will be adopted later. We construct such dummy variables identifying the median response to each answer and then identifying respondents above or at the median. In this way the dummy variables offer the partition of responses that is closest to splitting the observations in two evenly sized groups. Supplementary Table 6, Panel A, reports the results of a principal component factor analysis. We note that the eigenvalue for the first factor is fairly high (2.34) and can account for 59% of the total variance of the four items. A second eigenvalue is around unity and can explain an additional 24% of total variance. Although such a second factor would normally be considered weak and not retained, we nevertheless included it in the analysis of the factor loadings (Supplementary Table 6, Panel B). This shows that all four variables have a positive and rather strong loading on the first factor, ensuring the internal consistency of the construct 'Social Distance from BM'. Interestingly, the analysis of the second factor brings out that while 'Acquaintance with BM' and 'Acceptance of BM Guidance' have both a positive loading, both 'Frequency of Past Encounters' and 'Frequency of Future Encounters' have a negative loading. This supports our hypothesis that these two sets of variables capture different sub-dimensions of the more general 'Social Distance from BM' dimension.

A confirmatory factor analysis, reported in Supplementary Table 6, Panel C, further supports this claim. It contrasts two different models. Model 1 includes a unique latent variable ('Social Distance') which is assumed to be measured by all of the four variables of our questionnaire. Conversely, Model 2 includes two latent variables. One is associated with the 'Recognition of BM' dimension, which is measured by 'Acquaintance with BM' and 'Acceptance of BM Guidance'. The other, 'Social Connection', is measured by the frequency of encounters variables. We report five indicators of goodness of fit for the two models. Model 1 never satisfies any of the four tests of goodness of fit, while Model 2 satisfies all of them and scores better in an overall measure of fit – namely, the Coefficient of Determination (see note to Supplementary Table 6).

Finally, the Cronbach's alpha for the scale formed by the four variables altogether equals 0.76. This indicates that the reliability of the construct reaches acceptable levels. All of the four variables have a positive correlation with the scale and dropping any of them would reduce the reliability of the scale. The same can be said though for the two separate scales

formed by the first two items alone (Cronbach's alpha = 0.74) and the last two items (Cronbach's alpha = 0.84). We thus conclude that the factor analysis supports our hypothesis that overall the four questions measure a general dimension, which we called ´Social Distance from BM', and that this can be further broken down into two sub-dimensions ('BM Recognition' and 'Social Connection').

We now investigate whether our social distance measures can predict the cooperation rates observed in the experiment. We use a slightly modified version of the models used above, where a dummy variable identifying the two villages located in the hills replaces village dummies. We noted that cooperation rates tend to be higher in mountain villages than in coastal villages, and relatively little variability occurs within these two groups (see SI: Supplementary Discussion, Section 1.2). This variable, then, substantially controls for village heterogeneity and saves some degrees of freedoms in a regression with fewer observations than the previous ones. We purportedly do not include any dummy variables identifying the BM treatment against the BM EXT treatment, because we want to capture the whole variability of the increased social distance from the external Big Man in comparison with the local Big Man. At the same time, we introduce a dummy variable identifying the treatment BM+PUN to control for the fact that a different game was used in this treatment in comparison with the other two treatments. The model also includes controls for gender, experimenter identity, and comprehension. We do not consider additional demographic controls not to lose further observations due to missing values (see SI: Supplementary Discussion, Section 1.2).

We expect that of all the four variables are significantly and positively correlated with cooperation. On the one hand, the more an individual is acquainted with the Big Man and the more he is recognised as a legitimate guide for action, the stronger will be the individual's willingness to maintain a good social image in the eyes of the Big Man. On the other hand, the more an individual is closer to the Big Man in the social network, as reflected in their past and future frequency of encounters, the more the individual may feel exposed to the possibility of being sanctioned through, for instance, gossip (Dunbar and Dunbar, 1998; Sommerfeld et al., 2007).

The results are reported in Supplementary Table 7. The first four regressions introduce the four items one at a time in the basic model. The last four regressions include one variable of the "Recognition of BM' dimension and one of the 'Connection' dimension together. The main result of our analysis is that only the 'Connectivity' dimension is a significant predictor of cooperation, while the 'Recognition of BM' fails to predict cooperation. Neither

‘Acquaintance with BM’ (P=0.23) nor ‘Acceptance of BM guidance’ (P=0.76) have significant effects on cooperation. On the contrary, both ‘Frequency of Past Encounters’ (P=0.047) and ‘Frequency of Future Encounters’ (P=0.031) significantly predict cooperation. The more frequently an individual expects to meet the Big Man, the higher the probability of cooperation. When Connection variables are included in the same regression with ‘Acquaintance with BM’, they are either significant predictors of cooperation as for ‘Frequency of Future Encounters’ (P=0.048) or at the margins of significance for ‘Frequency of Past Encounters’ (P=0.078), while ‘Acquaintance with BM’ has no predictive power (P= 0.567 and P= 0.391). When Connection variables are matched with ‘Acceptance of BM guidance’, they are both significant predictors of cooperation (P=0.032 for ‘Frequency of Past Encounters’, P=0.024 for ‘Frequency of Past Encounters’), while ‘Acceptance of BM guidance’ has no predictive power (P= 0.560 and P= 0.815). Tests on the difference between the two coefficients of Acceptance of BM guidance’ is at the margins of statistical significance (P=0.095 and P=0.083).

These results suggest that the closeness between the participant and the Big Man in the social network, and the associated stronger possibility of sanctioning, matters more than the actual guidance that the BM can legitimately exert. This helps us qualify which aspect of one’s social image more at stake when a participant is observed by a BM. The exposure to informal retribution through more frequent contacts within the social network seems to matter more than the recognition of the BM authority in direct one-to-one interactions. This tentatively confirms, though indirectly, the relevance of informal social sanctioning such as gossiping in promoting cooperation.

1.4 Analysis of punishment

Summary Statistics:

Supplementary Table 8 reports summary statistics of punishment patterns in treatment BM+PUN and PUN broken down by altruistic punishment and anti-social punishment behaviour. Money spent for altruistic punishment is reduced by K0.71 (27.30 %) when the Big Man is present. According to the regressions presented in Supplementary Table 9, this reduction is statistically significant (P=0.019). Costs for anti-social punishment are reduced by K0.28 (14.29 %) when the Big Man is present. However, this reduction is not statistically significant (P=0.405).

In order to test our protocol in the context of a Western society, we replicated the study of the PUN condition at Kiel University in Germany, following as closely as possible the

original protocol (see SI: Supplementary Methods, Section 2.5). Supplementary Fig. 1 compares the percentage of defectors and 36o-operators who were punished in Teop and Germany. One can notice that only 8% of co-operators were punished in Germany (out of 37 participants), compared to 60% in Papua. The number of punished defectors is also considerably lower in Germany than Teop (32% against 72%). Similar results are found if we look at the fraction of one's endowment spent for punishment of defectors and 36o-operators. Our German sample spent 23% and 7% for the punishment of defectors and 36o-operators, respectively, which is substantially lower than what spent by participants in Teop (see Supplementary Table 8). The overall cooperation rate in Germany was 62%, which is close to what found in Teop (60%, see Supplementary Table 2). Therefore, the same levels of cooperation can be enforced with much lower punishment costs in Germany compared to Teop.

The results we obtained in Germany are consistent with other experiments conducted in Western Europe, and fit well with the cross-cultural analysis carried out by Gächter and Hermann (2010; 2009). More specifically, our results from Germany are equivalent to the results of one-shot PD with punishment conducted in Switzerland, which has been assigned to the same cultural area as Germany (Gächter and Hermann, 2010; 2011). We infer that nothing in our protocol induces an especially high level of punishment. Hence, the results obtained in Teop are likely due to the specific characteristics of the local population.

Supplementary Fig. 2 compares punishment patterns between the present study and the study of Gächter and Herrmann (2011) conducted in Russia. Since this study by Gächter and Herrmann (2011) includes a one-shot public goods game followed by a one-shot punishment stage, while most other studies include repeated interactions, it offers a suitable comparison to our study. It is remarkable that the punishment results in the Russian sample of Gächter and Herrmann (2011) are very similar to our punishment patterns. In Russia (Teop) 55% (60%) of the participants punished co-operators, while 75% (72%) of participants in Russia (Teop) punished defectors (see note to Supplementary Fig. 2 for the definition of anti-social and altruistic punishment in Gächter and Herrmann, 2011). This comparison supports the idea that anti-social punishment is widespread in many societies. Hermmann et al. (2008) find a significant correlation between experimental anti-social punishment and compliance with civic norms of good conduct in the society at large, as measured by cross-national surveys.

Regression analysis for punishment decisions:

Here we report the full econometric models for the regressions presented in the paper regarding punishment. The model reported in Supplementary Table 9: column 1, 5 and 9 include only treatment effects. In column 2, 6 and 10 we add village fixed effects as controls. Column 3, 7 and 11 include the following controls: village fixed effects, a dummy variable for experimenter identity effect, and comprehension. In columns 4, 8 and 12 we add further demographic controls. The variable 'Comprehension prisoner's dilemma' is based on the number of mistakes in the comprehension checks relative to the PD. All these variables have been used in the analysis of cooperation and are defined in SI: Supplementary Discussion, Section 1.2. Regression on Punishment further includes the variable 'Comprehension punishment stage', which is based on the number of mistakes to the comprehension questions relative to the punishment stage. This indicator measures participant's promptness of understanding payoff calculations in the punishment stage. Both 'Comprehension prisoner's dilemma' and 'Comprehension punishment stage' are coded in the following way: (0) no mistakes, (1) one mistake and (2) more than one mistake. In eight cases the comprehension variable was not available in the second stage. We thus replaced it with the comprehension measure for the PD game.

Results are reported in Supplementary Table 9. We note that demographic effects, as well as village effects, are in this case not significantly different from zero. The only significant effects are the higher level of total punishment and pro-social punishment in PUN compared to PUN+BM (see Supplementary Table 9, columns 1-8). The regressions in Supplementary Table 9, columns 3, 7, and 11 are those used to support the tests reported in Figure 2 of the article.

Supplementary Table 10 presents ordered logit regressions with specifications that control for religion. We report models that control for religious affiliation (Catholic or Protestant) and the frequency of attendances of religious services in the community (see SI: Supplementary Discussion, Section 1.2 for definition). While religious affiliation is not significantly correlated with punishment, low attendance of religious services correlates positively with both pro-social ($P = 0.026$, $N=109$; Supplementary Table 10, column 4) and anti-social punishment ($P = 0.010$, $N=109$; Supplementary Table 10, column 6). The effect on total punishment is therefore equally strong and significant ($P=0.002$; $N=218$; Supplementary Table 10, column 2).

1.5 Analysis of third party punishment

Summary statistics:

Supplementary Fig. 3 reports the mean fraction of endowment II (K4) spent by the third-party for altruistic punishment (punishing defectors) and anti-social punishment (punishing 38o-operators), taking into account the action performed by the PD-player's counterpart. Supplementary Table 11 presents summary statistics in more detail.

Regression Analysis

Here we report the full econometric models regarding third-party punishment. In order to allow for the full set of hypotheses testing on regression coefficients, we use an ordinary least square regression model instead of an ordered probit regression model.[1] The model reported in Supplementary Table 12: column 1 includes only treatment effects. In column 2 we add village fixed effects as controls. Column 3 includes the following controls: village fixed effects, a dummy variable for experimenter identity effect, and comprehension. We use Wald-tests – presented at the bottom of Supplementary Table 12 – to test 1) the null hypothesis that each coefficient of interest is different from zero and 2) the null hypothesis that coefficients of interest are equal to each other.

To analyse the monotonic trend between the amount of money possessed by the PD-player and the amount of punishment carried out by the third-party, in Supplementary Table 13 we define a new variable called PD-player payoff. This variable is based on the four payoff situations that can emerge in the PD and take on the values 0 (payoff of PD-player = K0), 1 (payoff of PD-player = K10), 2 (payoff of PD-player = K20) and 3 (payoff of PD-player = K40). The model reported in Supplementary Table 13: column 1 includes only treatment effects. In column 2 we add village fixed effects as controls. Column 3 includes the following controls: village fixed effects, a dummy variable for experimenter identity effect, and comprehension. This is the regression from which the correlation and the corresponding p-value reported in the paper are drawn.

1.6 Analysis of payoffs

Supplementary Fig. 4 reports the mean payoff per treatment. In the two punishment treatments (BM+PUN and PUN) we report the actual mean final payoff, i.e. the mean payoff from the PD game net of the expected costs sustained by both punisher and punished individuals. For these two treatments we also report the mean payoffs in the hypothetical situation in which all punishment costs were equal to zero, and show the fall in payoffs due to either anti-social punishment costs or pro-social punishment costs.

[1] Results are nevertheless robust to using orderd probit regression models.

Supplementary Table 14 includes Tobit regression models for expected net payoffs (columns 1 to 4), expected payoffs if anti-social punishment costs are set equal to zero (columns 5 to 8), and expected payoffs if all punishment costs are set equal to zero (columns 9 to 12). For each of these three groups, the first two models include either treatments effects only or treatments effects and village effects. The last two models include additional demographic controls, as per the models illustrated above (see Supplementary Tables 3 and 9). Expected payoffs are computed for each individual from the combination of her own actions and the mean behaviour in the treatment where the individual participated. 'Expected payoffs if anti-social punishment costs are set equal to zero' are computed setting to zero the costs associated with anti-social punishment for both punisher and punished. 'Expected payoffs if all punishment costs are set equal to zero' are computed setting equal to zero all costs for punishment suffered by both punisher and punished individuals. At the bottom of Supplementary Table 14 we also report the results of pairwise Wald tests of the null hypothesis that the coefficients of treatments dummies are equal to each other.

As noted in the article, payoffs in BM are strongly significantly higher than in the baseline condition and any other treatments. We note that final payoffs are significantly lower in PUN (K5.9) than BM+PUN (K8.9). The same holds comparing payoffs if anti-social punishment is set to zero (K5.9+K7.2=K13.21 for PUN compared to K8.9+K6.3=K15.2 for BM+PUN). Both differences are statistically significant in a Tobit model that controls for village effects, experimenter identity effects, gender and comprehension (see tests of linear combinations at the bottom of Supplementary Table 14, columns 1 to 4).

We note that even if anti-social punishment was somehow successfully "banned", payoffs in treatment BM+PUN and PUN would still be lower than the baseline. The difference is statistically significant for both PUN and BM+PUN treatments compared with baseline (see reported parameters in Supplementary Table 14, columns 5 to 8). We conclude that the introduction of punishment is overall detrimental, even if it was exclusively directed to punishing defectors.

If all punishments costs were equal to zero, payoffs would instead not differ significantly among each other (see tests on treatment differences at the bottom of Supplementary Table 14, columns 9 to 12). This latter result mirrors what found with respect to cooperation (see Supplementary Table 3).

Supplementary Methods

1 Description of Teop society

Teop is an Austronesian ethno-linguistic group located in the Northeast of the island of Bougainville, an autonomous region of Papua New Guinea (PNG) situated in the North Solomon Islands. It is one among 21 ethno-linguistic groups living on this island. The population of the region is 175,160 people (2000 census). Around 5000 people belong to Teop society (Lewis et al., 2015).

Teop residents live in villages that vary in size from 50 to 200 people and are either located along the coast or in the hills. Their subsistence is based on horticulture and pig husbandry, supplemented by fishing, hunting and foraging (Regan and Griffin, 2005). Gardening is the main subsistence activity, but hunting provides additional protein in their diets. Sweet potatoes and taro are primary staples. Most families raise chicken and pigs, which are also used as gifts in the exchanges and ceremonies recurrent in village life (e.g., weddings and reconciliation ceremonies to resolve social conflicts).

Some of the surplus from the subsistence sector is sold in markets. Small outdoor markets are frequently found along roads, where women and children sell their garden produce and other commodities such as string bags, betel nuts, and lime. Cocoa and copra are the main commercial crops. These crops are harvested several times per year and sold to intermediaries in the main market towns of the island.

Currently, there are few opportunities for engagement in wage labour on Bougainville except for government employment. From the 1970s until the end of the 1980s the Panguna copper mine was one of the biggest employers in PNG. During that time the mine brought thousands of non-Bougainvilleans to the island due to labour shortages. This immigration caused tensions among the Bougainvilleans (O'Callaghan, 2002). In 1990 these tensions, together with other factors, resulted in the outbreak of a civil war which lasted until 2001. At that time the whole island was affected by sanctions implemented by PNG government and allies. However, acts of warfare were mainly restricted to the central area of Bougainville.

Formal (centralized) institutions for the enforcement of legal rules and shared morality are largely absent in PNG. This means that social life is regulated almost exclusively by social norms (Bernhard et al., 2006). Teop is one of the societies of Polynesia/Melanesia whose social structure is organised around tribes and clans, and where these social norms are guarded by Big Men. Big Men have a large group of followers within their clan, related groups, and villages. They possess exclusive knowledge and “impose discipline, uphold the traditional way of life and give executive directions” (Cochrane 1970: 137). Social disputes or

problems of coordination between clans (wantoks) have traditionally been dealt with under the supervision or explicit intervention of Big Men. In this sense, Big Men have informal authority and act as “guardians of morality” within the society. Hence, they seem particularly salient figures with which to study individuals’ willingness to keep a “good reputation” in social interactions.

Another unique feature of Teop society is matrilineality. Women’s social position in Bougainville culture has its origin in land. The matrilineal kinship structure gives women considerable power over material resources and activities that are economically and ritually important. Women’s prerogative over land includes defining land boundaries, giving permission to hunt or to harvest timber, and the exclusive right to veto decisions on land-related matters. While male relatives have rights to ownership, their rights are limited and conditional on female relatives’ permission (Saovana-Spriggs 2003). Moreover, unmarried, divorced, or widowed brothers and sons reside in the homes of their female relatives.

Married men who live with their wife’s family are expected to spend a considerable amount of their time in their mother’s or sister’s household. Still, there are some predominantly male domains. Women seldom participate in politics (neither regional nor local) and do not physically take part in tribal or civil conflicts.

2 Experimental design, protocol and game procedures

The study protocol was approved by the Presidential Office of the Institute for the World Economy at Kiel University and the "Social and Behavioral Approaches to Global Problems" research area. Approval was also granted by the Regional Government of Bougainville and the Council of Elderly of Teop.

2.1 Sampling

Our fieldwork took place in October and November 2014 in eight villages located in the area of Teop. Two lead researchers from our team resided in the area for the duration of fieldwork. The first month was devoted to preparing the experimental script, selecting villages and making contacts with the village Big Men and piloting the experiment. The experimental sessions were run in the second month. Selected villages were required to have a minimal size of 80 people. Smaller villages normally include only few households, so selection from larger villages is necessary to make random matching across participants meaningfully convey the idea that they are being matched with a “stranger”. We also ensured that villages were sufficiently distant from each other to minimise the risk of “contagion” – i.e. participants speaking with other participants about the research, thus influencing new participants’ behaviour in the game. Prior to the conduction of the research sessions, our research team was

requested to give a presentation of our research methods and goals to the Big Men and, in some cases, to the group of elderly of the village. In all cases was our request granted approval. Our research project was also supported by the regional government of Bougainville and by the Teop Council of Elderly.

In each village we requested a Big Man with high authority and popular recognition to act as observer in some of the sessions. Although some examples of "Big Women" – i.e. "female Big Men" - exist, being a Big Man is predominantly a male prerogative. We preferred to invite exclusively male Big Men, in order not to introduce gender variation in our experiments. The external Big Man (see Table 1, main paper) was a native of Arawa, a town located approximately 30 miles south-east of Teop. The region of Arawa is inhabited by the Nasioi ethno-linguitstic group (Regan and Griffin, 2005). Unlike the Teop, the Nasioi speak a non-Austronesian language, which further accentuates the social distance between the local Big Men and the external one. The external Big Man communicated in Tok Pisin with participants. Big Men who acted as observers in the experiments received a monetary offer for their assistance. This is in accordance with local customs when research fieldwork is carried out in the area.

A total of 272 participants – 143 males and 129 females - voluntarily participated in 19 experimental sessions. Four more villages were later involved in a follow-up of the present study, whose goal was to study the effects of third party punishment (see SI: Supplementary Discussion, Section 1.5 and Supplementary Methods, Section 2.4). Participants were drawn from lists of village residents that were made available by the village chiefs. In order to attain a fully stratified sample, we recruited at least one member from each household residing in each village. The participant from each household was randomly drawn. In all villages the desired number of participants exceeded by some units the number of households. Therefore we randomly drew the few households from which a second member was asked to participate. People were invited to participate one or two days in advance and sometimes on the same day. They were asked to show up at a given time. If some people invited to participate were not available, we randomly recruited another person from the same household. In order to minimise collusion we allowed only small groups of people to be present in the research sessions. Each participant only took part in one session and one treatment. That is, our design is between-subject. Descriptive statistics of our sample are reported in Supplementary Table 1. We note that Kruskall-Wallis tests fail to reject the hypohesis that treatments are exogenous to the demographic characteristics of gender, age, education, and wealth. In other words, socio-demographic characteristics are evenly balanced across treatments.

Henrich et al. (2006, 2010) report that contagion - i.e. the transmission of information about the experiment from past participants to future participants, possibly indicating which actions to take in the game - and collusion – i.e. agreeing on a certain strategy prior to the participation in the game - represented a constant risk for their experimental fieldwork. For these reasons we planned to involve many villages in our fieldwork and we did not stay more than three days in each village. Once permission was granted by local Big Men to run our research, we gave notice as short as possible about when we would start recruitment and when sessions would be conducted. We warned the Big Man that in case of suspicion of collusion or contagion we would have left the village without paying the participants. Only in one village did we have a strong suspicion of contagion. This was the fifth village involved in our investigation. While average cooperation ranged between 40% and 60% in other villages, in this village only one cooperative decision and eleven defections in the Prisoner's Dilemma (PD) were recorded at the end of the first day.Some of the participants stated their preferred plan of action, i.e. keeping the 10 Kina (K10), even before the beginning of the explanation of the game. Comprehension quizzes were answered incorrectly with a higher frequency than what observed in previous villages, probably because participants could not detach themselves from the idea of not playing a defection strategy.

This occurrence is remarkably similar to what described in Henrich et al. (2010, Supplementary Information - SI henceforth-: p. 8). They too noted a dramatic change in behaviour in some villages (e.g., nearly all people playing the 50-50 split in an Ultimatum Game), with some players manifesting their decisions before the game had been explained. Like Henrich et al. (2006, 2010), we excluded the data from this village in our dataset, and we cancelled the planned second day of investigation. We suspect that this is an instance of contagion rather than collusion. Collusion should naturally lead to universal cooperation, which we never observed. The most likely explanation is that inhabitants from this village contacted participants from the village where the research had been conducted the day before (perhaps during a volleyball game that involved teams from the two villages, an extremely rare event). On the basis of this experience, we decided to cancel other sessions planned in this area and relocated our investigation to two other areas that are located a dozen miles to the South and to the North from the original area. We also took an additional measure of paying payoffs at the end of our stay in a village, rather than at the end of each day, to limit contagion within a village. We did not have any other suspicion of contagion in our sessions.

2.2 Randomisation of treatments

We run a standard PD in the baseline condition and four treatments (see Table 1, main paper). Our administration of treaments during sessions followed a pre-fixed order whose sequence was randomised prior to running the sessions. This randomisation was run independently for the two lead experimenters conducting the sessions, though it had some obvious constraints, e.g. a local Big Man could only be present in one session at a time. Another constraint was the impossibility of having an external Big Man assisting us throughout the duration of fieldwork. This was partly due financial constraints and by the inability of the external Big Man to stay away from his main residence for the full duration of the research. In fact, it was decided from the outset that the 'BM External' treatment (BM EXT) was to be seen mainly as a robustness check for the effect on individual behaviour that a non local Big Man could cause in comparison with the local'Big Man' (BM). As such, a lower number of observations was planned for BM EXT than other treatments. We run the BM EXT treatment only in three villages along five sessions. In some villages our sample capacity was lower because only one experimenter was present. In these cases we decided to drop one or two treatments in order to ensure a sufficiently statistical power at the village level. In all villages was the baseline condition administered. This gives us the possibility to consistently compare baseline cooperation levels across villages. In one village the entire set of five treatments were administered. In one village the punishment treatments were not administered while the three other treatments were. In four villages all treatments but BM EXT were administered. In two villages all treatments but BM and BM EXT were administered. The introduction of village dummies in the econometric analysis enables us to control for these imbalances in the administation of treatments across villages. Village effects are rare, but we do observe a tendency for villages located in the mountain area to be more cooperative than villages located on the coast (see SI: Supplementary Discussion, Section 1.2). No significant difference among villages emerged with respect to punishment.

2.3 Session procedures

Procedures closely followed those set out by Henrich et al. (2006) but departed from them in some important respects.

We recruited a group of five local collaborators to assist us in the preparation and the conduction of the research sessions. Three of them were responsible for the conduction of the interviews, patrolling the waiting area, and other organisational tasks. Two of them assisted the lead experimenters in the administration of the game. Since the lead experimenters were both males, we chose females assistants to balance the gender composition of the research

group in the game sessions. Only one participant at a time was present in the game session. The same assistants were present in all sessions. We recruited an additional group of two or three research assistants residing in the village where we conducted our research.They contacted the people selected to participate, assisted in the organisation of the session, and patrolled the experimental areas during the conduction of the sessions. All these people were recruited on a payment basis. Additional monetary compensations were given to other people for their services – e.g. preparing meals or renting rooms or other areas where we conducted the sessions.

Participants were summoned to a 'waiting area', and were assigned an ID-number by our local research assistants. Participants completed a questionnaire about socio-economic and demographic variables and received K5 in cash as a show-up fee prior to the experiment. Unlike Henrich et al. (2006), the game was never explained at this stage, in order to minimise the risk of collusion or contagion. While in the waiting area, participants were explained how payments would take place and how each individual would be matched with another anonymous individual. This was done by randomly drawing pairs of ID-numbers from all participants at the end of a session. People whose ID-numbers were drawn in the same pair would be matched with each other. This was visually explained to participants. It was thus unlikely that people present in the waiting area at the same time would be matched with each other. It was emphasised that payoffs would be handed out in opaque envelopes labelled with the ID-number that the participant had drawn at the registration. These were shown to the participants. Given participants' high illiteracy rates, all subjects agreeing to participate were asked to provide verbal consent. It was further emphasised that participants were free to discontinue participation in the research and leave the session any time they wished. In fact, however, no participant did so. In order to further protect their anonymity, participants were not required to sign any receipt and were said they could leave the research area as soon as they received their payments. This attempt to come close to a "double-blind" procedure was done to maximise participants' perception of privacy in making their choices, thus fostering revelation of truthful preferences. While in the waiting area, local assistants ensured that participants did not discuss any aspects of the research.

Participants were randomly assigned to a session conducted by either Lead Experimenter 1 (LE1) or Lead Experimenter 1 (LE2). LE1 and LE2 led the sessions in two separate 'playing areas', out of sight from each other (see Supplementary Fig. 7) with one participant at a time. In order to facilitate comprehension, the game was illustrated using a playing board and real money (see Supplementary Fig. 8). Participants' comprehension was tested asking them to

calculate payoffs corresponding to different pairs of actions. We recorded the number of mistakes in the comprehension questions, as well as the participant's gender and the duration of the session. In case of mistakes, the game was explained again and participants were asked to recalculate. Only participants who correctly answered two consecutive questions about individual payoffs in the four possible outcomes of the PD were allowed to take part in the game. Henrich et al. (2006) used the same criterion. Participants were invited to move money on the playing board as a way to show the experimenters the correct answer. In addition, participants in treatments PUN and BM+PUN had to correctly answer three questions relating to final payoffs under different punishment actions. This actually required stating four correct answers for the PD game and six more for the punishment stage. In total, we dismissed 24 participants (about 8% of the sample). A variable measuring the number of trials a participant had to go through before answering correctly is included in the econometric analysis but is not statistically significant.

After passing the comprehension stage, participants made their decisions inserting K10 bills into one of two envelopes. We decided to elicit cooperation rates in the simplest possible way, using a standard Prisoner's Dilemma (PD) game. People made the decision of either giving K10 to the other player or keeping K10 for themselves. This binary choice design is particularly appropriate for a subject pool who may be unfamiliar with algebraic computations and yet still allowed us to test cooperation attitudes in a meaningful way. One envelope was labelled "Give" and the other "Keep". Both envelopes were then to be inserted by the participants into a larger opaque envelope. This was done to protect the confidentiality of the choice. The K10 bills were tied to a stick (see Supplementary Fig. 8) so the experimenter could check by manipulating the large envelope that the K10 had indeed been inserted. Participants in the PUN and BM+PUN treatments made their punishment decision similarly.

We applied the strategy method for punishment decisions. Participants made two decisions assuming that their counterpart had either kept K10 or given K10. For each assumption they received three envelopes and one large envelope. The three small envelopes were labelled 'Spend K0', 'Spend K2' and 'Spend K4'. Participants indicated their punishment decision by placing a stick into one of the three envelopes. First, participants were asked to decide how much of their K4 endowment to spend to reduce their counterpart's payoff assuming this person had kept K10. Subsequently they were asked to decide how much money to spend assuming that their counterpart had given K10. We kept this order fixed throughout all sessions. We implemented a punishment ratio of 5:1. This is larger than the ratio normally implemented in the literature (Anderson and Putterman, 2006), but ensures that

defectors final payoffs - in case the other player cooperated –goes significantly below that of mutual cooperation if the defector is punished. It also enabled us to keep the explanation and the computation of payoffs in the game as simple as possible. Since the Kina currency has both K2 and K10 bills, we could visually show on the playing board that every time a K2 bill was spent by one player, the other player's money was reduced by K10.

The strategy method has the advantage of enabling us to collect information about punishment for actions that the other player did not actually choose. In our case this was also a necessity because at the time of making a choice the matching had not been carried out so we did not know the counterpart's choice. Whether the strategy method produces different results from the direct response method has been widely debated in the literature. The general conclusion is that the two methods yield qualitatively similar results (Brandts and Charness, 2000; Brosig et al., 2003; Oxoby et al., 2004; Falk et al., 2005) although the strategy method in some studies marginally decreases the magnitude of punishment relative to the direct response method (Brosig et al., 2003;. Falk et al., 2005). As the strategy method was implemented in both PUN and BM+PUN, its impact is constant across treatments.

Another important difference in our protocol from Henrich et al.'s (2006) protocol is that experimenters and assistants left the room when a participant made her decisions, while the Big Man stayed in the room and observed the choice in the relevant treatments. This was done to maximise the salience of the presence of the Big Man (rather than the experimenter) in relation to treatments where he was absent. Given the extreme simplicity of the game procedures, the risk of participants making a choice not corresponding to their wills was minimal. In fact, only one participant failed to make a meaningful choice in the PD, as she inserted the K10 stick in neither the 'Give' nor the 'Keep' envelope. The experimenter realised this mistake when opening the large envelope at the end of the session. The participant was called back and asked to make her decision again.

The Big Man was asked not to speak with participants while they were making their choices. The experimenters could in fact hear from outside the room whether the Big Man spoke to the participants, but in no cases did this occur. We also cautioned the Big Man against using gestures to influence participants' choices and we believe that we were successful in curtailing this activity for several reasons. First, no participants indicated that Big Men overtly tried to influence their choices. Second, and most convincingly. we believe that the only possible course of action that a Big Man might seek to promote would be mutual cooperation and refraining from punishment, since this would maximize resources accruing to the community. However, our data show patterns of behavior that are far from this in all

villages studied. Third, before beginning the research we stated very clearly that in any case in which we observed a suspicious pattern in participants' choices (e.g., collusion), we would abandon the village and cancel the remaining sessions. We believe that this "threat" of abandoning sessions in a village, and the consequent loss of income for villagers, gave sufficient incentives to the Big Man not to interfere with participants' choices. As reported in Section 2.1, in one instance we did indeed leave a village after the first day; in this village we observed almost universal defection, regardless of the presence of the Big Man.

All sessions were conducted in Tok Pisin, the PNG lingua franca, by LE1 and LE2. Both LE1 and LE2 are fluent Tok Pisin speakers. They read instructions from a written script and followed a fixed protocol in order to ensure consistency of the procedures. LE1 and LE2 also observed each other delivering instructions in some pilots of the experiment. In some cases limited parts of the instructions were repeated in the Teop language by the local assistant to ensure full comprehension. All of the econometric analyses include a dummy variable controlling for experimenter identity effects, which is never statistically significant.

2.4 Additional third-party sessions

After conducting the main experiment we ran some additional experimental sessions involving third-party punishment (hereafter TPP). The main reason for this was to test whether anti-social punishment persisted in a game where the punisher was not involved in the PD. Anti-social punishment carried out by PD-players may be driven by aversion to disadvantageous inequality. In TPP games this can be excluded by design.

As above, the TPP game had two decision stages. In the first stage, two players played a PD in exactly the same way as in the baseline condition (see SI: Supplementary Methods, Sections 2.3 and 3.2). Prior to playing the game, they were informed that two other players – the third parties - would independently participate in a second stage of the game. Third parties were endowed with K34 each by the experimenters. Of this endowment, K30 were not used in the game and was paid to the third party. This ensures that the third party's payoff can never be less than any PD-player's payoff. K4 could be spent to reduce one of the two PD-players' payoffs, with costs being identical to the punishment costs in the PUN and BM+PUN treatments. That is, the third party could spend either K0, K2 or K4 to reduce the PD-player payoff by K0, K10, or K20, respectively. Each third party could only punish one PD-player. The third party was asked to make four decisions through the "strategy method", one for each of the four possible outcomes of the PD. A total of 25 participants took part in the PD and 21 participipnats acted as third parties. To compensate for this disparity between second and third

parties, we used twice the decisions of four randomly drawn third parties, and applied their decisions to the second parties.

2.5 Additional second-party punishment sessions in Germany

In order to test our protocol in the context of a Western society, we replicated the study of the PUN condition at a University in Germany, following as closely as possible the original protocol. We focused on the PUN treatment because it would have been difficult to find a design corresponding to treatments involving Big Men in Teop. We were particularly interested in running sessions with punishment, rather than the baseline, to ascertain the extent of anti-social punishment in a Western society in the context of our protocol.

Participants were recruited via flyers and posters that we hung in libraries, cafes and restaurants. We wanted to reach out to an adult sample, rather than university students, for increased comparability between Teop and Germany. The average age of the German sample is 38 years (S.D.: 13.1, Minimum =18; Maximum = 61) and 54% of the sample has educational attainment below a university degree. The gender composition is rather unbalanced as only 35% of the German sample are females. Unlike in Teop, where sessions were conducted individually, in Germany sessions were run collectively, including between 5 to 10 people at a time. After signing up for the research, participants were invited to attend a session at a given time slot in a university room. They were seated at different tables, with enough distance between them to guarantee privacy. The PD and the punishment stage were explained to the whole group with the help of audio-visual support, which resembled the "playing board" used for instructions in Teop. After delivering the instructions, participants were asked to answer comprehension questions before they made their decisions. Checking comprehension individually, and possibly repeating instructions to those participants failing to answer correctly, would have unduly extended the length of each research session, which we promised would last around half an hour. For this reason we preferred to record the number of correct answers to the comprehension check initially and in a second trial (in case some mistakes were made). We only include in the ensuing analysis the 37 participants who answered all comprehension questions correctly at the end of the first or the second trial. The experiments were run with pen and paper. Rather than handling real money as in Teop, participants had to indicate on a sheet their preferred actions. In total we conducted eight sessions. We did not aim to equalise the purchasing power of the money at stake between Germany and Teop, but used the same numerical stakes, playing in Euros (€) rather than Kina. Participants in Germany were therefore initially assigned 10€, which would be doubled in case the player gave the 10€to the other player. People could spend 0€, 2€, or 4€ to punish

the other player. The 5:1 ratio of money deducted versus money spent on punishment was maintained as in Teop.

For reference, we note that one litre of petrol and one Kilo of rice cost K5 and K3.95 in Teop, respectively. At the exchange rate current at the time the research was conducted (1 Kina per 0.32€), this is equivalent to 1.6€ and 1.26€, respectively. The same goods could be purchased in Germany at the price of 1.38€ per litre of petrol and and 1.5€ per Kilo of rice. This shows comparable levels of purchasing power for the two currencies with respect to goods that are bought on international markets. Macroeconomic data nevertheless reveal a large per capita GDP gap between the two countries equal to 2,112€ in Papua New Guinea and 35,120€ in Germany.

2.6 Payments

Although most of the Teop are not employed in formal labour markets and do not receive salaries, money is commonly used in economic transactions. It seemed therefore appropriate to pay participants in the local currency rather than using other sources of value, such as for instance rice or sugar. We applied monetary incentives comparable to Henrich et al. (2006). Interviews run prior to the fieldwork ascertained that the average daily wage rate in Teop was K20 (equivalent to 6.4€). We then designed the monetary incentives to be the same as those given by Henrich et al. (2006). Accordingly, the show-up fee of K5 was 20% of the daily wage rate, and the final payoffs in case of mutual cooperation equalled the daily wage rate. The K5 show-up fee was explained to participants as their compensation for participating in the interview preceding the game and was handed out at the end of registration and interview by local researchers. The payment of the game payoff was done either at the end of each session or when leaving the village after two or three days. This latter method was implemented after the first two weeks both to maximise the time devoted to conducting experiments during the day and to minimise contagion effects (see SI: Supplementary Methods, Section 2.1). Handing out payments at a fixed time after making the choice is of course the best way to ensure that time discounting effects did not influence participants' choices. Nevertheless, we do not find any qualitative differences between villages where the two methods were implemented. Variables measuring the time of the day when a participant made her decision, and the day at which researchers were active in a village are never statistically significant in our econometric analyses, which suggests that contagion effects were successfully contained.

As previously mentioned, payoffs were handed out in opaque envelopes labelled with the ID-number that the participant had drawn at registration. Participants were encouraged to

check their payoffs privately, and in no instance were the experimenters able to identify the payoffs earned by specific participants.

3 Experiment Protocol and game instructions

3.1 Pre-Experiment instructions

The following instructions were read by the local research assistant individually to subjects in the waiting area (WA=Waiting area; PA = playing area; LE1=Lead Experimenter 1; LE2=Lead Experimenter 2; RA1 = research assistant 1, presiding over WA, helped by other research assistants from our team or from the village; RA2=research assistant 2; assisting LE1; RA3 = research assistant 3, assisting LE2).

Hello! My name is [RA1] and I am here with [LE1] and [LE2] to conduct this study. They are researchers from the University of [Name University where LE1 and LE2 are affiliated]. Thank you for taking the time to come today and taking part in it. Here you have 5 Kina (K5) for your participation and for the interview. This is your money. I cannot take it back again. Take them and put it away in some safe place, they will not be needed for the decisions you will make later on.

Ok, this survey is about how people make decisions. Before we begin I want to make some general comments about what we are doing here today and explain some rules that we need to follow. By making decisions in the following game you can earn real money that you will take home. This money is yours to keep. But maybe you won't get any money. There is no right or wrong decision and you cannot lose any money. You should understand that this is not my own money and it is not [LE1's] or [LE2's] money. It is money given to them by their university to use to do a research study. The money will be paid in cash when the sessions are finished. You should come here with this card that you have just drawn and your payments will be given to you in an envelope. Please remember to take this number with you when you come to collect your payment.

Before we proceed any further, let me stress something that is very important. Many of you were invited here without understanding very much about what we are planning to do today. If at any time you find that this is something that you do not wish to participate in for any reason, you are of course free to leave whether we have started the game or not. If you decide to do so, please inform us and return to us the research materials. Let me tell you something about the decisions you are going to make.

Decisions are made by two individuals, you and another person. This person may be from this village, or from another village from this area. Before coming here we run this research in other villages as well. None will know with whom you are matched.

We have invited many people from this area to take part in this game. At the end of the sessions, we will take your decision and match it randomly with the decision of another participant.

You will be making decisions with these envelopes here. You will receive your money in this envelope named "My Earnings". Now I write your number on this envelope and on all other envelopes. The other person also receives his money in an envelope like this one.

Now let us explain how we pair the two persons who will make a decision. At the end of the session we will draw two envelopes at a time at random from the "Decisions" box. These two people will be matched, and their decisions will determine their earnings. [Show an example of matching. Do NOT show content of envelopes]

For instance, here I draw envelope # X and envelope # Y. So, participant # X and participant # Y will be matched together and their decisions will determine their earnings.

You will be explained the decision later in the room over there. Please wait your turn quietly here. Everyone caught talking about the game before or after he participates will not receive any money! After you finish the game, you may not enter the WA again under any circumstances. If you do so, we may have to cancel the session and leave this village without handing out any payment. [When a PA is free, RA1 invites the participant to go to that PA.]

3.2 Individual instructions in the experimental session

3.2.1 Script of the Game: Prisoner's Dilemma

Hello and thank you for you coming here. My name is LE1/LE2. I hope you can understand my Tok Pisin. If not, I will ask our assistant to translate. I am here with [RA2/RA3] {Treatment BM, BM+PUN: and [observer's name] who will help me in this study.}. {Treatment BM, BM EXT, BM+PUN: [observer's name] is a Big Man in {Treatment BM, BM+PUN: this village}; {Treatment BM EXT: Arawa} .} [Big Man introduces himself, "My name is…, I come from {Treatment BM, BM+PUN: this village}; {Treatment BM EXT: Arawa}, and I am here to assist LE1/LE2 in this research.".]

Now I explain the game with this board. {PUN and BM+PUN: There are two parts in this game. I start explaining the first part.} It is important that you understand well, because only people who understand the game well will take part in it. Moreover, what you will earn depends on the decision you make, so you must understand well. You will receive your

earnings inside this envelope “My earnings”. [Put envelope “My earnings” by participant’s side. Also point to envelope “Other person’s earnings”.]

Decisions are made by two individuals, you and another person. The other person is not here now but he or she will make a decision in exactly the same way as what you are going to do. Perhaps he or she has already made a decision. This person may be from this village, or from another village from this area. Before coming here we run this research in other villages as well. The other person, too, will receive his money inside an envelope similar to yours.

Ok, now I give you K14. The other person also receives K14. The K10 I give the other person in this board have a green stick. {Baseline, BM, BM EXT: These K4 will not be used in the game, and will be paid out to you when you come to collect your payments inside your “My Earnings” envelope. Now I put this K4 aside and you can forget about it for the rest of the game.} [Put the K4 in the envelope marked “”My Earnings”. Do the same for other participant.] {BM+PUN, PUN: These K4 will be used later in the second part of this game so I put them aside. Let’s now see the first part of this game}. [Put the two K2 bills aside. Put aside K10 belonging to other person. Put K20 bills towards the middle of the board, and say that this is your (i.e. the researcher’s) money.].

Now I tell you what you can do with your K10. You can make one out of two things. You can either keep these K10 or give these K10 to the other person. If you keep the K10, these K10 are yours. They will be put inside your “Earnings” envelope [Show K10 going into Earnings envelope]. If you give the K10 to the other person, I will add K10 and pass them on to the other person. So the other person receives K20 while you are left with nothing. I will put these K20 inside the “Earnings” envelope of the other person. [Move the K10 bill across the line and add another K10]

The other person has the same choice as you. [Remove subject’s own K10 from board and put green K10 onto other person’s side]. If the other person keeps the K10, these K10 are his and I will put them inside his “Earnings” envelope. If he gives the K10 to you, I will add K10 and pass them on to you so that you end up with K20 while the other person is left with nothing. I will put these K20 inside the “Earnings” envelope of the other person. [Move the other person’s K10 bill across the line and add another K10]. So, every time a person gives K10, I add K10 and the other person receives K20. [Show again for both giving and receiving K10.]

After each Person makes the decision to keep or give the money {Baseline, BM, BM EXT: the game is over.}; {PUN, BM+PUN: the first part of the game is over.}

Ok, let us check if you are clear about the game. I am going to show you some examples. You will tell me how much money you and the other person will get. You can use the board to move the money and add K10 to calculate the payoffs. [All the examples below should be visualized on the board, placing the two envelopes “My Earnings “Other Person’s Earnings” at the top end of the table. If more test questions are needed the researcher or assistant should again start from the first example].

Example Nr.1: You and the other person both keep the K10. How much money would you get inside your envelope at the end of this example? How much does the other person have? [K10; K10] Ok. So you both have K10 in this situation. Now the next example.

Example Nr. 2: You keep your K10 and the other person gives his K10 over to you. How much money would you and the other person have inside your envelope at the end of this example? [K30; K0] Ok. So you have K30 and the other person has nothing in this situation. Now the next example.

Example Nr. 3: You give your K10 over to the other person, and the other person keeps his K10. How much money would you and the other person have inside your envelope at the end of this example? [K0; K30] Ok. So you have nothing and the other person has K30 in this situation. Now the next example.

Example Nr. 4: You and the other person both give the K10 to each other. How much money would you and the other person get inside your envelope at the end of this example? [K20; K20] Ok. So you both have K20 in this situation.

[Give time to answer each question and explain the correct answer if the participant gives the wrong answer. If participant does not answer correctly all questions, the game is explained again and the participant is asked to answer again. The number of mistakes in the first set of questions is recorded. In the subsequent checks, participants should answer correctly the first question and at least another question. If they still make mistakes, participants are said that they cannot take part in the game.]

{Baseline, BM, BM EXT: Now we have finished with the explanations of the game. Do you have any questions?}

{BM, BM+PUN: Now we have finished with the explanations of the first part of the game. Do you have any questions?}

3.2.2 Script of the Game: Punishment stage {Treatments BM, BM+PUN only}

Ok, let’s now come to the second part of this game. After you have made your first decision, you will make two more decisions that will affect how much money you and the

other person take home. [Show second board. Leave first board to its left, placing K20 on both players' sides on the first board.].

For this second part you use the K4 that we left aside from the first part. [Place the two K2 bills in front of both players on the second board.] Let us suppose that both you and the other person have K20 at the end of the first decision. [Put K20 at the other person's side on first board]. In this second part you can spend some money to reduce the other person's earnings. If you pay K2 I will reduce by K10 the money that the other person will take home. [Show on board]. If you pay K4 I will reduce by K20 the money that the other person will take home. [Show on board]. If you pay nothing, I will not reduce the other person's money. [Show on board].

So, you can do one thing out of three things: Option number 1: You can keep your K4. Option number 2: You can keep K2 and spend K2. I reduce the other person's earnings by K10; Option number 3: You can keep nothing, spend K4, and I will reduce the other person's earnings by K20. [Show three envelopes with three options].

Remember that the other person has the same 3 options: Option number 1: He can keep his K4. Option number 2: He can keep K2 and spend K2. I reduce your earnings by K10; Option number 3: He can keep nothing, spend K4, and your earnings will be reduced by K20.

Remember, the money you spend and the money I take away will go outside the game; you will not receive this money nor the other person will receive your money. [Show on board, using 2-kina sticks for other person and 10-kina belonging to subject.]

Is this clear? Ok, I would like you to answer what is the correct answer to the following examples:

Example No. 1: Suppose that the other person has K20 after the first decision. Suppose you decide to spend 0 kina. How much money do I take away from the other person? [Solution: None.]

Example No. 2: Suppose the other person has K10 after the first decision. You decide to spend K2. How much money do I take away from the other person? [Solution: K10].

Example No. 3: Suppose that you have K30 after the first decision. The other person decides to spend K4. How much money do I take away from you? [Solution: K20].

Example No. 4: Suppose that you have K10 at the end of the first decision. The other person decides to spend K4 to reduce your earnings. What are the final earnings for you?

Ok, this is a special situation. The other person wants to reduce your earnings by K20, but you only have K10. In all situations where one person wants to reduce the other person's

earnings by an amount bigger than what that person has, I take away all the money that that person has, but nothing more. In this case I take away your K10.

So, the money you will take home at the end of the session is: how much money you have from the first part, minus how much money the other person takes away from you, plus the money you keep from these K4.

Example No. 1: You and the other person have K20 at the end of the first part. The other person takes K10 away from you. You spend K2 to take away K10 from the other person. How much money do you take home? [Ask participant to answer.] In this case you both take home K12.

Example No. 2: You have K30 at the end of the first part. The other person has no kina. The other person spends K2 and takes away K10 from you. You spend nothing. How much money do you and the other person take home? [Ask participant to answer.]

In this case you take home K24. The other person takes home K2.

Example No. 3: You and the other person have both K10 at the end of the first part. The other person takes no money away from you. You take no money away from the other person. [Ask participant to answer.] In this case you take home K14.

Ok, now I add an important thing: You will make two decisions in this second part. One decision is for when the other person keeps the K10 in the first part. The other decision is for when the other person gives you K10 in the first part. At the end of the session I will see what the other person has done. So I take away money or not take away money from his according to your decision.

3.2.3 Explanation of how to make decisions in Part I

Now you will make your decision {BM+PUN, PUN: in the first part of the game}. I give you two envelopes, one called “Keep” and the other called “Give”. If you want to keep the K10, you will have to place the K10 into this envelope. If you want to give the K10, you will have to place the K10 into this envelope. [Show envelopes.]

While you make your decision, I will not be at this table so we will not watch your decision. {BM, BM EXT, BM+PUN: but [observer’s name] will be here and watch your decision}. }. {Baseline, PUN: You make your decision alone.}. When you are finished, please put your two envelopes “Keep” and “Give” into this big envelope. [Show decision envelope.] If it is all clear, you can make your {BM+PUN, PUN: first} decision now. [RA2/RA3] and I will go out {BM, BM EXT, BM+PUN: while Mr [observer] will stay here}. Please call us

when you are finished. [After decision has been made] Ok, now I am going to put your decision inside the Decision box.

3.2.4 Explanation of how to make decisions in Part 2{BM+PUN, PUN only}:

Now you will make the decisions for the second part of the game. Let’s first consider the situation when the other person keeps the money in the first half of the game. Here these three envelopes represent your three possible options. [Show on first board that K10 are above “Other person’s earnings” envelope.] You have to put this stick inside the envelope that matches what you want to do. [Put the stick inside the 3 envelopes. Illustrate 3 envelopes]. After you have made your decision, you will put the three envelopes into this big “Decision” envelope. [RA2/RA3] and I will go out {BM+PUN: while Mr [observer] will stay here}. Please call us when you are finished. [Come back when subject has finished.] Ok, now I am going to put your decision inside the Decision box.

[Explain and repeat for envelope “Person 2 gives”. In this case put K20 by the subject’s side. Say:] Let’s now consider the situation when the other person gives you the money. In this case the other person gave you K10, so here are K20 going to your payment envelope. Here these three envelopes represent your three possible options. [RA2/RA3] and me will go out. {BM+PUN: while Mr [observer] stays here}. Please call us when you are finished.

At the end of the session, LE1/LE2 thank the participant and give instructions about payoff collection. In treatments BM, BM EXT and BM+PUN, RA2/RA3 privately ask four questions about social distance with the Big Man (see SI: Supplementary Discussion, Section 1.3).

3.3 Additional script: Third-Party Punishment

Instructions of the first stage were as in every PD session. The instructions below followed to explain the punishment stage.

3.3.1 Explanation of punishment stage to PD-Players

Ok, let’s now come to the second part of this game. After you have made your first decision, a third person will make a decision that may affect how much money you and the other person take home. [Show a second playing board. Leave first board to its left, placing K20 on both players’ sides on the first board. Second board should have “Person Number 3 and Person Number 4” clearly written on the horizontal sides].

We will call the person who makes the decision in this second part the ‘third person’. This person is NOT the person with whom you have made the previous decision. This is another person who made her decisions in the previous days. You and the other person from

the first part don’t have to make any decisions here. [Place the two K4 in front of both players on the second board.]

Let us suppose that both you and the other person have K20 at the end of the first decision. [Put K20 at the other person’s side on first board]. Person 3 receives some money from me. He can then spend K4 to take away some money from you and the other person. The third person can make one thing out of three things: The third person can spend K2 to subtract K10 from the money you will earn at the end of the day; or the third person can spend K4 to subtract K20 from the money you will earn at the end of the day; or the third person can spend nothing, i.e., the third person keeps the money and leave your earnings unchanged. Let’s see on the board how this works. Let´s suppose that you have K20 from the first part of this decision.

So, the third person can do one thing out of three things. Option number 1: The third person can keep K4. Option number 2: The third person can keep K2 and spend K2 to take K10 away from you. Option number 3: The third person can keep nothing, spend K4, and take K20 away from you.

Ok, now I add an important thing: The third person will make four decisions in this second part. One decision is for when both you and the other person keep K10 in the first part. One decision is for when both you and the other person give K10 in the first part. One decision is for when you keep K10 and the other person gives K10 in the first part. The last decision is for when you give K10 and the other person keeps K10 in the first part. At the end of the day I will see what you do and take away money or not take away money from you according to the third person’s decision.

Remember, the money that I take away will go outside the game; the third person will not receive this money, nor the other person will receive this money.

Ok, now I add another important thing. There is another fourth person in the second part. This person will receive also money from me. The fourth person can then spend money to take away some money from the person you are matched with in the first part of the game. The fourth person makes the same four decisions in the second part like the third person does. [Show on board, using K2-sticks for third person and K10 belonging to “Other person”.]

Is this clear? Ok, I would like you to answer what is the correct answer to the following examples. [Show on board, using K2-sticks for third person and K10 belonging to subject.]

Example No. 1: You and the other person have both given K10 and you have K20 at the end of the first part. Person 3 decides to spend K2 when both of you give K10. How much money do you take home? [Solution: K10].

Example No. 2: Suppose you have K10 after the first decision. The third person decides to spend K2. How much money do I take away from you? [Solution: K10].

Example No. 3: Suppose that you have K30 after the first decision. The third person decides to spend K4. How much money do I take away from you? [Solution: K20].

Example No. 4: Suppose that you have K10 at the end of the first decision. The third person decides to spend K4 to reduce your earnings. What are the final earnings for you? [Solution: K0]

Ok, this is a special situation. The other person wants to take away K20 from you, but you only have K10. In all situations where one person wants to take away more money than another person has, we take away all the money that that person has. In this case I take away your K10.

So, the money you will take home at the end of the day is: how much money you have from the first part, minus how much money the third person takes away from you.

3.3.2 Explanation of PD and how to make decision in the punishment stage for third-party

Hello and thank you for you coming here. My name is LE1/LE2. I hope you can understand my Tok Pisin. If not, I will ask our assistant to translate. I am here with [RA2/RA3].

Now I explain the game with this board. There are two parts in this game. I start explaining the first part. It is important that you understand well, because only people who understand the game well will take part in it. Moreover, what you will earn depends on the decision you make, so you must understand well. You will receive your earnings inside this envelope “My earnings”. [Put envelope “My earnings” by participant’s side. Also point to envelope “Other person’s earnings”.]

Decisions in the first part are made by two individuals, which we call Person 1 and Person 2. These two people are not here now. They will make their decisions in the next days in exactly the same way as what you are going to do. These people may be from this village, or from another village from this area.

Person 1 and Person 2 will receive K10 each. [Put the 10 Kina onto the two sides of the board.] Person 1 and Person 2 can make one out of two things. Each of them can either keep these K10 or give these K10to the other Person. If Person 1 keeps the K10, these K10 are hers. They will be put inside her “Earnings” envelope. [Show 10 kina going into Earnings envelope] If Person 1 gives the K10 to the other person, I will add K10 and pass them on to

the other Person. So the other person receives K20 while Person 1 is left with nothing. [Move the 10 Kina bill across the line and add another 10 Kina] -

Person 2 has the same choice as Person 1. [Remove subject's own 10 Kina from board and put green 10 kina onto other person's side.] If Person 2 keeps the K10, these K10 are hers and I will put them inside her "Earnings" envelope. If she gives the K10 to Person 1, I will add K10 and pass them on to Person 1 so that Person 1 ends up with K20 while the Person 2 is left with nothing. I will put these K20 inside the "Earnings" envelope of the other person. [Move the other person's 10 Kina bill across the line and add another 10 Kina]

So, every time a person gives K20, I add K10 and the other person receives K20. [Show again for both giving and receiving 10 kina.]

After each Person makes the decision to keep or give the money, the first part of the game is over.

Ok, let us check if you are clear about the game. I am going to show you some examples. You will tell me how much money Person 1 and Person 2 will get. You can use the board to move the money and add K10 to calculate the payoffs. [All the examples below should be visualized on the board, placing the two envelopes named "My earnings" "Other person's earnings" at the top end of the table]. If more test questions are needed the researcher or assistant should again start from the first example.]

Example No. 1: Person 1 and Person 2 both keep the K10. How much money would they get inside their envelopes at the end of this example? [K10; K10]

Ok. So they both have K10 in this situation. Now let's see the next example.

Example No. 2: Person 1 keeps her K10 and Person 2 gives her K10 over to Person 1. How much money would they have inside their envelopes at the end of this example? [K30; K0]

Ok. So Person 1 has K30 and Person 2 has nothing in this situation. Now let's see the next example.

Example No 3: Person 1 gives her K10 over to the other person, and the other person keeps her K10. How much money will they have inside their envelopes at the end of this example? [0 Kina; 30 kina].

Ok. So Person 1 has nothing and Person 2 has K30 in this situation. Now let's see the next example.

Example No 4: Person 1 and Person 2 both give the K10 to each other. How much money would you and the other person get inside their envelopes at the end of this example? [K20; K20] Ok. So they both have K20 in this situation.

[Give time to answer this question. If payoffs are unclear, go through the examples again and check. If payoffs still unclear, turn the subject away without paying the extra kina. They should answer correctly the first question with no help, and at least another questions even with one cue. If they don't get one question right with at least one giving go through 4 examples again.]

Now we have finished with the explanations of the first part of the game. Do you have any questions?

Explanation of punishment stage

Ok, let's now come to the second part of this game. Now you will make a decision that may affect how much money Person 1 and Person 2 take home. [Show second board. Leave first board to its left, placing 20 kina on both players' sides on the first board. Second board should have "Myself" clearly indicated.]

Now I give you K34. K30 are not used in the game and are for you to keep. You will receive them in your payoff envelope at the end of the game.

[Insert K30 inside the "My Money" envelope.]

Let us suppose that both Person 1 and Person 2 have K20 at the end of the first decision. You can spend some of your money to take away some money from Person 1. You can spend K2 to subtract K10 from Person 1's money; or you can spend K4 to subtract K20 from Person 1's money. Or you pay nothing and don't take away money from Person 1. [Show on board]

So, you can do one thing out of three things: Option number 1: You don't spend any money and keep all of your K34. I don't take away money from Person 1. Option number 2: You can spend K2. I take away K10 from Person 1; Option number 3: You can spend K4, and I take K20 away from the other person. [Show three envelopes with three options].

Remember, the money that I take away will go outside the game; you will not receive this money, nor the other person will receive this money.

Another important thing: there will be another person who, like you, receives K34. This person can spend money to take away money from Person 2, like you did for Person 1.

Is this clear? Ok, I would like you to answer what is the correct answer to the following examples. [Show on board, using 2-kina sticks for third person and 10-kina belonging to subject]

Example No. 1: Suppose that both Person 1 and Person 2 have given. They have K20 after the first part. Suppose you decide to spend K0. How much money does Person 1 take home? [Solution: 20 kina] [They should answer correctly this example, leading answer is OK. If they do, just show second example without requiring answers. Otherwise request answer.

Example No. 2: Both Person 1 and Person 2 have kept their K10 in the first part. They both have K10 at the end of the first part. You spend K2. How much money does Person 1 take home? [Solution: K0]

Example No. 3: Person 1 has kept her K10 while Person 2 has given her K10 to Person 1. Person 1 has K30 at the end of the first part and Person 2 has nothing. You spend K4. How much money does person 1 take home? [Solution: K10] [Require answer from Example 3.]

Example 4: Person 1 and Person 2 have held back their K10. Both Person 1 and Person 2 have K10 at the end of the first part. You spend K4. How much money does Person 1 take home? [Solution: K0]

Ok, this is a special situation. You want to take away K20 from Person 1, but Person 1 has nothing. In all situations where one person wants to take away more money than another person has, I take away all the money that that person has, but nothing more. In this case I take away your K10.

Ok, now I add an important thing: You will make four decisions in this second part, for each possible choice that Person 1 and Person 2 can make. The first decision is for when Person 1 and Person 2 both keep the K10 in the first part. The second decision is for when Person 1 keeps K10 and Person 2 gives K10. The third decision is for when Person 1 and Person 2 both give K10. The fourth decision is for when Person 1 gives K10 and Person 2 keeps K10.

Now I don't know what the other players have done. So I am asking you to tell me what to do for each possible case. When I know their decisions, I will take away money or not take away money from Person 1 according to your decision.

Now you will make the decisions for the second part of the game. Let's first consider the situation when Person 1 and Person 2 both kept K10 in the first part of the game. Both have K10. [Put K10 at each side of the first playing board, in proximity of "Person 1" and "Person 2" writing]. Here these three envelopes represent your three possible options. How much do you want to spend, K0, K2 or K4? You have to put this stick inside the envelope that matches what you want to do. [Put the stick inside the 3 envelopes. Illustrate 3 envelopes]. After you have made your decision, you will put the three envelopes into this big "Decision" envelope. [RA2/RA3] and I will go out. Please call us when you are finished. [LE1/2 and RA2/3 both leave the playing area. They come back when subject has finished.] Ok, now I am going to put your decision inside the Decision box.

Let's now consider the second situation when Person 1 kept K10 and Person 2 gave K10. Person 1 has K30 and Person 2 has K0 after the first part of the game. [Put K30 near the

"Person 1" writing and no money by the side of "Person 2"]. Here these three envelopes represent your three possible options. How much do you want to spend, K0, K2 or K4? You have to put this stick inside the envelope that matches what you want to do. [Put the stick inside the 3 envelopes. Illustrate 3 envelopes]. After you have made your decision, you will put the three envelopes into this big "Decision" envelope. [RA2/RA3] and I will go out. Please call us when you are finished. [LE1/2 and RA2/3 both leave the playing area. They come back when the participant has finished.] Ok, now I am going to put your decision inside the Decision box.

Let's now consider the third situation when Person 1 gave K10 and Person 2 gave K10. Person 1 has K20 and Person 2 has K20 after the first part of the game. [Put K20 at both sides of the first playing board, in proximity of "Person 1" and "Person 2" writing]. Here these three envelopes represent your three possible options. How much do you want to spend, K0, K2 or K4? You have to put this stick inside the envelope that matches what you want to do. [Put the stick inside the 3 envelopes. Illustrate 3 envelopes]. After you have made your decision, you will put the three envelopes into this big "Decision" envelope. [RA2/RA3] and I will go out. Please call us when you are finished. [LE1/2 and RA2/3 both leave the playing area. They come back when the participant has finished.] Ok, now I am going to put your decision inside the Decision box.

Let's now consider the fourth situation when Person 1 gave K10 and Person 2 kept K10. Person 1 has nothing and Person 2 has K30 after the first part of the game. [Put no money by the side of "Person 1" in the first playing board and K30 by the side of "Person 2]. Here these three envelopes represent your three possible options. How much do you want to spend, K0, K2 or K4? Please note that Person 1 has no money at the end of the first part. Therefore, for any amount of money you will spend, I will not take away any money from Person 1. You have to put this stick inside the envelope that matches what you want to do. [Put the stick inside the 3 envelopes. Illustrate 3 envelopes]. After you have made your decision, you will put the three envelopes into this big "Decision" envelope. [RA2/RA3] and I will go out. Please call us when you are finished. [LE1/2 and RA2/3 both leave the playing area. They come back when the participant has finished.] Ok, now I am going to put your decision inside the Decision box. You have made all decision now and the session is over.

Supplementary References

Anderson, Christopher M., and Louis Putterman. "Do non-strategic sanctions obey the law of demand? The demand for punishment in the voluntary contribution mechanism." *Games and Economic Behavior* 54.1 (2006): 1-24.

Bernhard, Helen, Urs Fischbacher, and Ernst Fehr. "Parochial altruism in humans." *Nature* 442.7105 (2006): 912-915.

Brandts, Jordi, and Gary Charness. "Hot vs. cold: Sequential responses and preference stability in experimental games." *Experimental Economics* 2.3 (2000): 227-238.

Brosig, Jeannette, Joachim Weimann, and Chun-Lei Yang. "The hot versus cold effect in a simple bargaining experiment." *Experimental Economics* 6.1 (2003): 75-90.

Cameron, A. Colin, and Pravin K. Trivedi. *Microeconometrics: methods and applications*. Cambridge university press, 2005.

Cochrane, Glynn. *Big men and cargo cults*. Clarendon Press, 1970.

Dunbar, Robin, and Robin Ian MacDonald Dunbar. *Grooming, gossip, and the evolution of language*. Harvard University Press, 1998.

Falk, Armin, Ernst Fehr, and Urs Fischbacher. "Driving forces behind informal sanctions." *Econometrica* 73.6 (2005): 2017-2030.

Gächter, Simon, and Benedikt Herrmann. "The limits of self-governance when cooperators get punished: Experimental evidence from urban and rural Russia." *European Economic Review* 55.2 (2011): 193-210.

Gächter, Simon, Benedikt Herrmann, and Christian Thöni. "Culture and cooperation." *Philosophical Transactions of the Royal Society of London B: Biological Sciences* 365.1553 (2010): 2651-2661.

Henrich, Joseph, et al. "Costly punishment across human societies." *Science*, 312.5781 (2006): 1767-1770.

Henrich, Joseph, et al. "Markets, religion, community size, and the evolution of fairness and punishment." *Science* 327.5972 (2010): 1480-1484.

Herrmann, Benedikt, Christian Thöni, and Simon Gächter. "Antisocial punishment across societies." *Science* 319.5868 (2008): 1362-1367.

Huber, Peter J. "The behavior of maximum likelihood estimates under nonstandard conditions." *Proceedings of the fifth Berkeley symposium on mathematical statistics and probability*. Vol. 1. No. 1. 1967.

Kish, Leslie, and Martin Richard Frankel. "Inference from complex samples." *Journal of the Royal Statistical Society. Series B (Methodological)* (1974): 1-37.

Lewis, M. Paul, Gary F. Simons, and Charles D. Fennig (eds.). *Ethnologue: Languages of the World.* SIL International, 2015.

O'Callaghan, M. *The origins of the conflict* in Carl, A. and L. Garasu, (eds.) Weaving Consensus: The Papua New Guinea-Bougainville Peace Process. Conciliation Resources, 2002.

Oxoby, Robert J., and Kendra N. McLeish. "Sequential decision and strategy vector methods in ultimatum bargaining: evidence on the strength of other-regarding behavior." *Economics Letters* 84.3 (2004): 399-405.

Regan, A. and Griffin, H. (eds.) (2005). *Bougainville Before the Crisis*. Canberra: Pandanus Books.

Saovana-Spriggs, Ruth. "Bougainville women's role in conflict resolution in the Bougainville peace process." *A Kind of Mending: restorative justice in the Pacific islands, Pandanus Books, Canberra* (2003): 195-213.

Sommerfeld, Ralf D., et al. "Gossip as an alternative for direct observation in games of indirect reciprocity." *Proceedings of the National Academy of Sciences* 104.44 (2007): 17435-17440.

White, Halbert. "A heteroskedasticity-consistent covariance matrix estimator and a direct test for heteroskedasticity." *Econometrica: Journal of the Econometric Society* (1980): 817-838.

Woolridge, J.M. *Introductory Econometrics: A Modern Approach.* South-Western College Publishing, 2002.
